# Innate immune imprints in SARS-CoV-2 Omicron variant infection convalescents

**DOI:** 10.1038/s41392-022-01237-y

**Published:** 2022-11-16

**Authors:** Zhiqing Li, Xiaosu Chen, Junyan Dan, Tianju Hu, Ye Hu, Shuxun Liu, Yangyang Chai, Yansong Shi, Jian Wu, Hailai Ni, Jiaqi Zhu, Yanfeng Wu, Nan Li, Yizhi Yu, Zhongfang Wang, Jincun Zhao, Nanshan Zhong, Xianwen Ren, Zhongyang Shen, Xuetao Cao

**Affiliations:** 1 National Key Laboratory of Medical Immunology, Institute of Immunology, Naval Medical University, Shanghai, 200433 China; 2 Frontier Research Center for Cell Response, Institute of Immunology, College of Life Sciences, Nankai University, Tianjin, 300071 China; 3 Department of Immunology, Institute of Basic Medical Research, Peking Union Medical College, Chinese Academy of Medical Sciences, Beijing, 100005 China; 4 The Health Care Department, Shanghai Changhai Hospital, Shanghai, 200433 China; 5 Department of Cardiology, Shanghai Changhai Hospital, Shanghai, 200433 China; 6 Guangzhou Laboratory, Guangzhou, 510300 China; 7 Changping Laboratory, Beijing, 102206 China; 8 Organ Transplant Center, Tianjin First Central Hospital, Nankai University, Tianjin, 300192 China

**Keywords:** Innate immunity, Infection, Infectious diseases

## Abstract

SARS-CoV-2 Omicron variant infection generally gives rise to asymptomatic to moderate COVID-19 in vaccinated people. The immune cells can be reprogrammed or “imprinted” by vaccination and infections to generate protective immunity against subsequent challenges. Considering the immune imprint in Omicron infection is unclear, here we delineate the innate immune landscape of human Omicron infection via single-cell RNA sequencing, surface proteome profiling, and plasma cytokine quantification. We found that monocyte responses predominated in immune imprints of Omicron convalescents, with IL-1β-associated and interferon (IFN)-responsive signatures with mild and moderate symptoms, respectively. Low-density neutrophils increased and exhibited IL-1β-associated and IFN-responsive signatures similarly. Mild convalescents had increased blood IL-1β, CCL4, IL-9 levels and *PI3*^+^ neutrophils, indicating a bias to IL-1β responsiveness, while moderate convalescents had increased blood CXCL10 and IFN-responsive monocytes, suggesting durative IFN responses. Therefore, IL-1β- or IFN-responsiveness of myeloid cells may indicate the disease severity of Omicron infection and mediate post-COVID conditions.

## Introduction

The coronavirus disease 2019 (COVID-19) pandemic caused by the severe acute respiratory syndrome coronavirus 2 (SARS-CoV-2) has been going on for more than 2 years, resulting in great public health hazards.^1^ The SARS-CoV-2 Omicron variant has rapidly become dominant after its emergence.^2,3^ The immune profile of previous variants of SARS-CoV-2 has been studied in great depth by single-cell sequencing, but not enough for Omicron variant.^4–7^ Our previous research on the Omicron outbreak in Tianjin and other studies revealed that pre-infection vaccination reduced the severity of COVID-19, causing asymptomatic to moderate symptoms.^8–11^ However, Omicron variant has been reported being capable of escaping from recognition by neutralizing antibodies elicited by vaccination or previous infection.^12–14^ Furthermore, the T-cell response to Omicron spike proteins has been shown to be reduced by more than 50% in the vaccinated or previously infected individuals.^15^ Therefore, adaptive immunity is insufficient to explain the reduction in the severity of COVID-19 disease caused by Omicron variant. Immune mechanisms beyond adaptive immunity may function effectively in the protection against Omicron infection. It is reported that trained immunity, referring to “memory” innate immunity induced by infection or vaccination, is critical for immune defenses to unrelated invading pathogens.^16,17^ It is of urgent need to illustrate how innate immunity contributed to the disease progression of Omicron infection.

Following acute COVID-19 infection, a proportion of convalescents develop post-acute sequelae of COVID-19 (PASC) or Long COVID, with persistent symptoms including but not limited to fatigue, dyspnea, chest pain, loss of taste or smell, neuropsychiatric disturbances, arthralgia and decline in quality of life.^18,19^ Inflammation, organ and tissue injury, and/or coagulation during acute phase of COVID-19 have been supposed to be key drivers of PASC or Long COVID.^20^ The persistent inflammation in convalescents may be consequences of aberrant innate immune responses and chronic inflammation.^21^ However, the underlying pathophysiology from innate immunity in convalescents with PACS or Long COVID is still poorly understood.

We herein recruited 143 COVID-19 convalescents who had experienced mild to moderate disease after Omicron infection and applied single-cell RNA sequencing targeted to human immune response genes (TTA scRNA-seq), single-cell whole-transcriptome sequencing (WTA scRNA-seq), surface proteome profiling of 30 immune cell-associated antigens (Abseq), and plasma cytokine/chemokine measurements to comprehend the immune imprints of Omicron infections. Our results illustrate that monocytes and neutrophils exhibited distinct immune imprinting patterns, with IL-1β-associated and interferon (IFN)-responsive signatures in patients with mild and moderate symptoms, respectively. We also identified a novel *PI3*^+^ neutrophils subpopulation with more frequencies in mild convalescents. In addition, we identified a group of *EGR1*^+^ monocytes which can differentiate into pro-inflammatory *CCL3*^+^ monocytes, indicating the potential sources of systemic inflammation in COVID-19 patients. Furthermore, monocytes and cytokines with IFN-responsiveness were associated with post-acute sequelae of COVID-19, implying that IFN responses may be important for modulating post-COVID conditions.

## Results

### Plasma cytokine and chemokine profile of Omicron convalescents

**Fig. 1:**
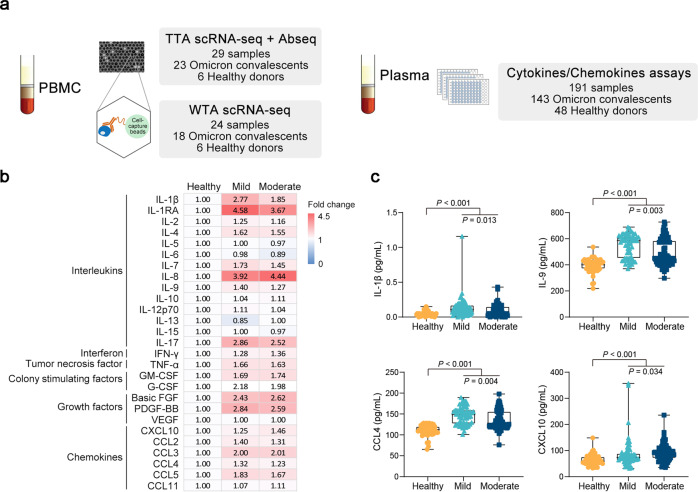
Schematics of the overall study design and plasma levels of cytokines and chemokines in SARS-CoV-2 Omicron convalescents. **a** Schematics depicting the overall experimental design of this study. Peripheral blood mononuclear cells (PBMCs) and plasma were collected from SARS-CoV-2 Omicron convalescents and matched healthy controls and then processed to single-cell RNA sequencing (scRNA-seq), surface proteome profiling (Abseq), and cytokine and chemokine assays. **b**, **c** Plasma levels of cytokines and chemokines from SARS-CoV-2 Omicron convalescents (*n* = 143) and healthy controls (*n* = 48). SARS-CoV-2 Omicron-infected individuals were divided into mild (*n* = 62) and moderate (*n* = 81) disease severity groups according to WHO living guidance for clinical management of COVID-19. Twenty-seven cytokines and chemokines were measured by the Bio‐Plex pro human cytokine assays and MAGPIX system (Luminex). Fold change of average plasma concentrations of cytokines and chemokines were shown in (**b**), and the differences in cytokines and chemokines between mild and moderate convalescents were shown in (**c**). Statistical significance was determined by two-tailed Mann–Whitney U test

To characterize the immunological features of COVID-19 patients infected by SARS-CoV-2 Omicron, we collected peripheral blood from 143 Omicron convalescents who had experienced mild or moderate COVID-19 and 48 age- and sex-matched healthy donors. The plasma and fresh peripheral blood mononuclear cells (PBMCs) were obtained ~42 days after infection for cytokine and chemokine assays and single-cell level expression profiling, respectively. PBMC specimens from 23 convalescents and 6 healthy donors were used for TTA scRNA-seq and Abseq, of which 18 convalescents and 6 healthy donors’ specimens were used for WTA scRNA-seq, summarized in Fig. 1a and Supplementary Table. S1–S3.

To evaluate the immune characteristics of the Omicron BA.1-infected convalescents, the levels of cytokines and chemokines in the plasma of convalescents and matched healthy donors were first measured. Seventeen of twenty-seven cytokines and chemokines were higher in convalescents plasma than in healthy controls, including IL‐1β, IL‐1RA, IL-4, IL-7, IL-8, IL-9, IL-17, TNF‐ɑ, GM-CSF, G-CSF, basic FGF, PDGF‐BB, CXCL10, CCL2, CCL3, CCL4 and CCL5 (Fig. 1b). Notably, convalescents who experienced mild symptoms had higher levels of IL‐1β, IL-9, and CCL4 but lower level of CXCL10 than moderate ones (Fig. 1c and Supplementary Fig. 1a–c). After outlier exclusion, the trend and significance remains (Supplementary Fig. 2a–c).

Together, Omicron-infected individuals in convalescence still presented with innate immune activation to some extent, and IFN-γ-inducible protein CXCL10 was prominently abundant in the plasma of convalescent patients who had experienced moderate symptoms, while IL-1β in mild convalescents.

### Single-cell atlas of the peripheral blood immune profiles in Omicron convalescents

To characterize the immune properties of convalescents from the Omicron infection, we performed microwell-based TTA scRNA-seq and Abseq analysis of fresh PBMCs using the Rhapsody system. After filtering out low-quality, duplex and low-viable cells, a total of 413,299 high-quality single cells were ultimately obtained. All data from 29 donors were integrated into a comparable dataset after correction for batched effects.

**Fig. 2:**
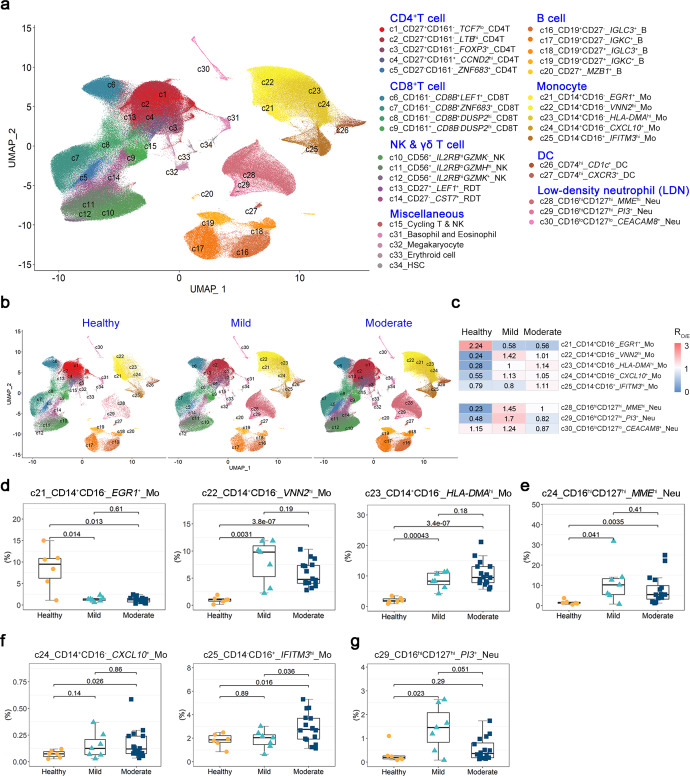
The peripheral immune cell profiling of the Omicron variant-infected convalescents and healthy donors. **a** UMAP plots showing all the 34 cell clusters in peripheral blood from convalescents with mild to moderate Omicron infection and healthy controls. Clusters were named based on the cluster-specific expression patterns at protein and transcript levels, followed by the major cell type. See also Supplementary Fig. S3, S4 and Table. S4. **b** UMAP plots showing the cell clusters in PBMCs from the Omicron convalescents experienced mild or moderate clinical symptoms and healthy controls. **c** The composition of monocytes and low-density neutrophils (LDNs) measured by the ratio of observed to randomly expected cell numbers (R_O/E_) in PBMCs from the Omicron convalescents (*n* = 7 for mild and *n* = 16 for moderate) and healthy controls (*n* = 6). **d** The composition of monocytes measured by percentages in PBMCs from the Omicron convalescents and healthy controls. *t*-test with Welch’s correction. **e** The composition of LDNs measured by percentages in PBMCs from the Omicron convalescents and healthy controls. *t*-test with Welch’s correction. **f** The composition of monocytes measured by percentages in PBMCs from the moderate convalescents and healthy controls. *t*-test with Welch’s correction. **g** The composition of LDNs measured by percentages in PBMCs from the mild convalescents and healthy controls. *t*-test with Welch’s correction

We obtained 34 cell clusters based on the expression of 397 immune profile genes at transcript levels. Surface proteome targeting cell type hallmarks and functions by Abseq sequencing was also utilized to better define each cell cluster (Supplementary Fig. S3a). We discriminated these cell clusters by surface or intracellular hallmarks and genes with different expression abundance and named them in the order of surface antigens at protein levels and then genes at transcript level followed by major cell types. In these clusters, CD4^+^ T, CD8^+^ T, NK cells, monocytes, T_γδ_ cells and low-density neutrophils (LDNs) contained different subsets or distinct status (Fig. 2a and Supplementary Fig. S4a, Table. S4). LDNs were isolated together with PBMCs during density gradient centrifugation, and are reported abnormally increased in autoimmune diseases, cancer and acute viral infections including COVID-19 patients.^22–24^ Based on the expression of *LEF1*, *TCF7*, *LTB*, *DUSP2*, *CST7* and cytotoxic molecules, we discriminated T cells and NK cells at resting or naive status (*LEF1*^+^*TCF7*^lo^), activated status (*LTB*^hi^*DUSP2*^hi^*CST7*^hi^), effector or memory (*ZNF683*^+^), stem-like (*CCND2*^+^) and cytotoxic types of cytotoxic cells (*GZMK*^+^ or *GZMH*^+^). We discriminated CD14^+^ monocytes at naive or steady (*EGR1*^+^), regulatory (*VNN2*^hi^), and antigen-presenting-associated (*HLA-DMA*^*hi*^) states by the expression of *EGR1*, *VNN2*, *HLA-DMA*. LDNs were defined by the distinct expression levels of *MME*, *PI3* and *CEACAM8* (Supplementary Fig. S4a, b). In summary, TTA-scRNAseq integrated with surface proteome profiling presents an extraordinary heterogeneous landscape of innate and adaptive immunity.

To ascertain the cellular source of those increased plasma cytokines and chemokines in convalescents, we performed an overview of the cellular distribution at the transcript level of all detectable cytokines and chemokines. Only 17 cytokines and chemokines could be detected at transcript levels, including *IL-32*, *CCL5*, *CCL4* and *IFNG* mainly from T and NK cells, *CXCL16*, *IL1B*, *IL1RN*, *CCL3*, and *CXCL8* mainly from monocytes and LDNs, *VEGFA* mainly from DC and monocytes, *IL-18* mainly from DC and HSC, *CXCL10* and *CXCL2* exclusively from the *CXCL10*^+^ and *EGR1*^+^ monocyte subsets respectively, and *CXCL5* only from megakaryocytes (Supplementary Fig. S4b). *IL-9*, *IL-4*, *IL-7*, *IL-17*, *G-CSF*, *GM-CSF*, *CCL2*, *CCL11*, *PDGF* and *FGF* were undetected in any clusters of PBMCs, implying that these serum cytokines might be produced from tissue-resident immune cells or non-immune cells.

### The impacts of Omicron infection on innate immunity in convalescents

To understand the imprinted immunity by Omicron variant infection, we first analyzed the effects of the Omicron infection and the COVID-19 severity on the compositions of all cell clusters. We found that it had a profound impact on the cellular compositions of both innate and adaptive immunity in convalescents (Fig. 2b and Supplementary Fig. S5a–c). CD14^+^*HLA-DMA*^hi^ and CD14^+^*VNN2*^hi^ monocytes, and CD4^+^CD27^+^CD161^-^*LTB*^hi^ T cells were higher in both mild and moderate convalescent patients than in healthy donors according to ratios of observed to randomly expected cell numbers (R_O/E_) and percentages in PBMCs calculated for each cell cluster. Consistently, naive or steady state cells including CD14^+^*EGR1*^+^ monocytes and *LEF1*^hi^*TCF7*^lo^ CD4^+^ T cells were both lower in mild and moderate convalescents than in healthy donors (Fig. 2c, d and Supplementary Fig. S6a, b). Additionally, these convalescents displayed increased LDNs and megakaryocytes (Fig. 2c, e and Supplementary Fig. S6a, b), suggesting recruitment from bone marrow. Collectively, these results revealed that these convalescents still maintained a certain degree of immune responsive status irrespective of the severity of the disease.

**Fig. 3:**
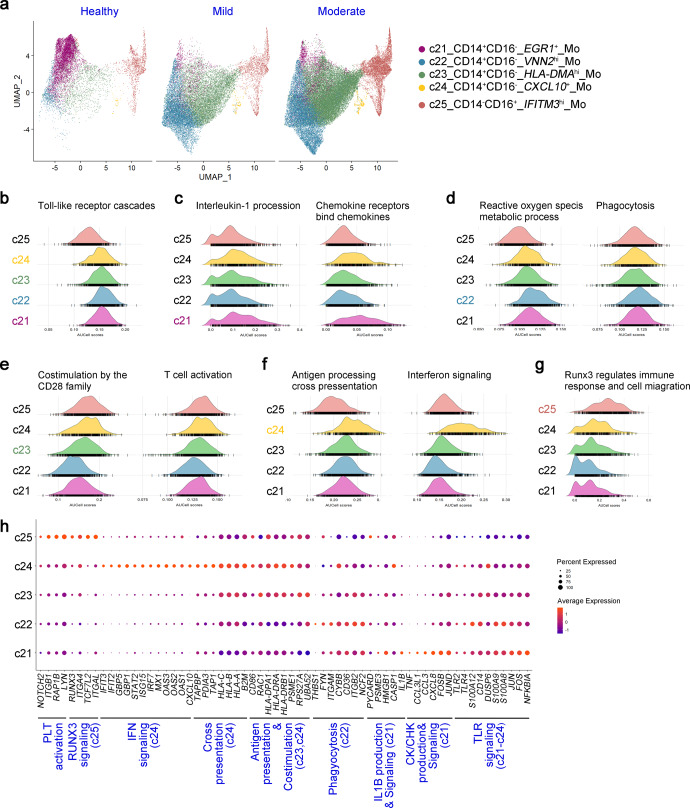
Biological processes and signaling pathways enriched by the monocyte clusters in Omicron convalescents. **a** UMAP plots showing the distribution of all the monocyte clusters in convalescents after mild to moderate Omicron infection (*n* = 5 for mild and *n* = 13 for moderate) and healthy donors (*n* = 6). **b** AUcell plots showing the enrichment on TLR-mediated cascades by all monocyte clusters. **c** AUcell plots showing the enrichment on the production and responses to the signaling pathways of inflammatory cytokines and chemokines by all monocyte clusters. **d** AUcell plots showing the enrichment on the reactive oxygen species production and phagocytosis by all monocyte clusters. **e** AUcell plots showing the enrichment on the processes of antigen-presenting, T cell and B cell activation by all monocyte clusters. **f** AUcell plots showing the enrichment on the processes of IFN-mediated signal pathways by all monocyte clusters. **g** AUcell plots showing the enrichment on the responses to RUNX3-mediated immune response signal pathways by all monocyte clusters. **h** Dot plot depicting the expression of genes involved in the immune processes and signaling pathways enriched above by these monocyte clusters

With respect to the disease severity and immune responsive status, mild convalescents had more CD127^+^*PI3*^+^ LDNs than healthy individuals and moderate convalescents, while moderate convalescents had more CD14^+^*CXCL10*^+^ monocytes than healthy donors and more CD14^-^CD16^+^*IFITM3*^hi^ monocytes than healthy donors and mild convalescents (Fig. 2c, f, and g). Both CD14^+^*CXCL10*^+^ monocytes and CD14^-^CD16^+^*IFITM3*^hi^ monocytes highly expressed classical interferon-responsive genes including *IFITM3, OSA1, IRF7, MX1, etc*. (Supplementary Fig. S4b and Fig. 3h), indicating durable IFN-responsive status in innate immunity in moderate convalescents. As expected, the expression of *IFITM3* was prominently higher in moderate convalescents than in mild ones and healthy individuals (Supplementary Fig. S6c). Additionally, naive or resting-status *LEF1*^+^ T_γδ_ cells were less in moderate convalescents than in healthy ones, while *ZNF683*^+^CD4^+^ CTL cells were higher in moderate convalescents than in mild ones (Supplementary Fig. S6a, d, e), suggesting a persisting CD4^+^ CTL response in convalescents. Altogether, the composition of certain myeloid cell clusters (CD16^+^*IFITM3*^+^ monocyte and *PI3*^+^ LDN) at responsive status varies with the disease severity.

### Monocytes exhibit dichotomous immune imprints associated with IL-1β- and IFN-responses in Omicron convalescents

We further extracted monocytes to fully depict the heterogeneity of CD14^+^CD16^−^ and CD14^−^CD16^+^ monocytes in all healthy and convalescent individuals (Fig. 3a). We analyzed immune response-associated processes and signal pathways enriched by each monocyte cluster based on the data of WTA scRNA-seq of 48,181 monocytes.

All the four CD14^+^ monocyte clusters exhibited comparable levels in response to Toll-like receptor (TLR) cascades, while the CD14^−^CD16^+^ monocytes were less TLR-responsive, indicating that classical CD14^+^ monocytes were the major population in response to TLR-dependent external and endogenous stimuli (Fig. 3b, h). The *EGR1*^+^ monocytes displayed the highest responsiveness to the signaling pathways of cytokines such as IL-1β, IL-10, chemokines and the production of IL-1β and chemokines, by the high expression of transcripts implicating in initiating or acting downstream of their signaling pathways, such as *FOS*, *FOSB*, *JUN*, *JUND*, *etc*. (Fig. 3c, h and Supplementary Fig. S7a). The *EGR1*^+^ monocytes were the predominant monocytes in healthy donors, indicating that they were “ready-to” cells with high potential in response to signals from TLR agonists and inflammatory stimuli.

The other three CD14^+^ monocyte clusters were responsive monocytes dominated in convalescents. *VNN2*^hi^ monocytes were superior to the other clusters in producing reactive oxygen species, TSP1 pathway and particulate exogenous antigen phagosomes, due to highly expressing *NCF2* (necessary for active oxygen generation), complement receptor 3 (*ITGAM*/*ITGB2*), *CD36*, *FYN* and *THBS1* which mediate phagocytosis (Fig. 3d, h and Supplementary Fig. S7b). Compared to other monocyte clusters, *VNN2*^hi^ monocytes were less functional for antigen-presenting, co-stimulating T cell activation and B cell activation (Fig. 3e and Supplementary Fig. S7c). By contrast, the *CXCL10*^+^ and *HLA-DMA*^hi^ monocytes had high expression of MHC-II genes, *CD74* and costimulatory molecule such as *CD86* (Fig. 3h). *CXCL10*^+^monocytes were characterized by IFN-responsive and cross-presentation function, with high expression of several IFN-responsive genes and genes in MHC I-restricted cross-presentation (Fig. 3f). CD14^+^*CXCL10*^+^ monocytes were the only cellular source of CXCL10 in peripheral blood (Supplementary Fig. S7d), which was elevated in moderated convalescents compared to healthy donors.

CD14^−^CD16^+^ monocytes also showed IFN-responsiveness, but to a less extent as compared to CD14^+^*CXCL10*^+^ monocytes (Fig. 3f). RUNX3 and NOTCH-mediated pathways were enriched in CD16^+^ monocytes, indicating that the differentiation of these non-classical monocytes was dependent on the signaling pathways of RUNX3 and NOTCH. Additionally, these CD16^+^ monocytes demonstrated high activity of EPHA4 pathway compared to those CD14^+^ monocytes, which play a role in the interaction with platelets (Fig. 3g, h and Supplementary Fig. S7e).

Considering that TLR and IL-1β-mediated pathways generally stimulate the production of active oxygen species and upregulate the expression of MHC-II and costimulatory molecules, we termed these clusters as IL-1β-responsive populations. The *VNN2*^hi^ monocytes and *HLA-DMA*^hi^ monocytes demonstrated less IFN-responsive signatures. Additionally, we also found that plasma IL-1β level was reversely correlated with the expression of *IFNGR1* in monocytes (Supplementary Fig. S7f). Additionally, these monocyte clusters were also present in COVID-19 convalescents infected by the ancestral strain according to published scRNA-seq datasets, and the enrichment signaling pathways were analogical (Supplementary Fig. S8a–d). In summary, CD14^+^ and CD16^+^ monocyte clusters exhibited distinct preferences to IL-1β- or IFN-responsiveness in convalescents.

### Low-density neutrophil subtypes with distinct IL-1β or IFN-responsiveness and protective *PI3*^+^ neutrophils in Omicron convalescents

**Fig. 4:**
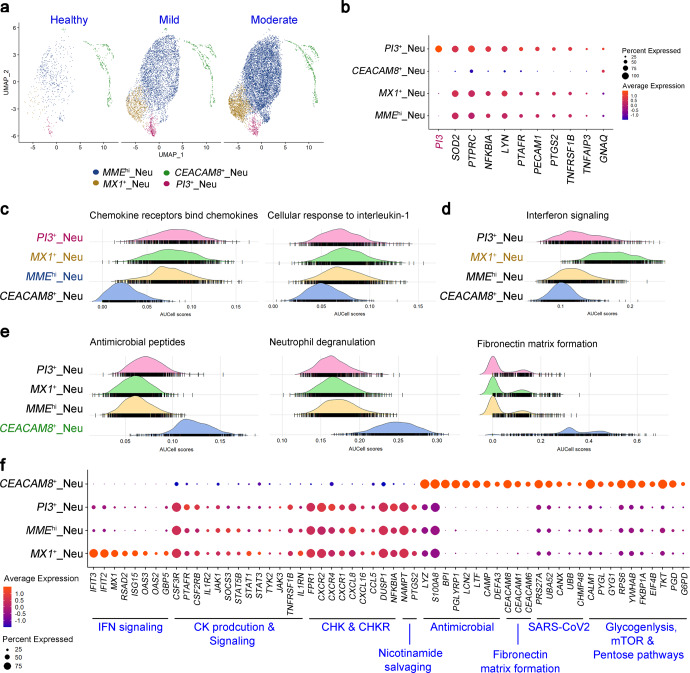
Biological processes and signaling pathways enriched by the low-density neutrophil (LDN) clusters in Omicron convalescents. **a** UMAP plots showing the cell clusters of LDNs from convalescents after mild to moderate Omicron infection (*n* = 5 for mild and *n* = 13 for moderate) and healthy donors (*n* = 6) using data of WTA scRNA-seq. **b** Dot plot depicting the differential expression genes by all the LDN clusters. **c** AUcell plots showing the enrichment on the production and responses to the signaling pathways of inflammatory cytokines and chemokines by all the LDN clusters. **d** AUcell plots showing the enrichment on the cell response to IFN pathway by all the LDN clusters. **e** AUcell plots showing the enrichment on the processes of innate immune defense and tissue remodeling by all the LDN clusters. **f** Dot plot depicting the expression of genes enriched on the biological process and signaling pathways described above by the LDN clusters

We also analyzed LDNs by scRNA-seq of the fresh PBMCs. To better understand the features of these LDNs in Omicron convalescents, we extracted data of LDNs from WTA scRNA-seq with the same UMI as LDNs by TTA scRNA-seq for subsequent biological process and pathway enrichment analysis. The original *MME*^hi^ LDNs by TTA scRNA-seq included two subclusters, of which one subcluster highly expressing many IFN-responsive genes such as *MX1*, *ISG15*, and *IFIT2*,^25,26^ so termed as *MX1*^+^ LDNs (Fig. 4a, f and Supplementary Fig. S9a, b). Specifically, we identified *PI3*^+^ LDNs undescribed in previous studies (Fig. 4b). PI3 is a serine proteinase inhibitor with an important role in protecting tissues from excessive injury during inflammatory events.^27,28^ *PI3*^+^ LDNs had higher frequencies in the convalescents with mild symptoms (Fig. 2c and Supplementary Fig. S9c). Additionally, the three clusters of *MME*^hi^, *PI3*^+^, *MX1*^+^ LDNs all highly expressed protective and inhibitory molecules such as *SOD2*, *NFKBIA* and *TNFAIP3*, *etc*., compared to the *CEACAM8*^+^ LDNs, with *PI3*^+^ LDNs expressed higher levels than *MX1*^+^ and *MME*^hi^ LDNs (Fig. 4b), indicating that these LDNs were more biased to immune regulatory and protective nature than *CEACAM8*^+^ LDNs.

*MME*^hi^, *MX1*^+^ and *PI3*^+^ LDNs exhibited higher production of inflammatory cytokines and chemokines, similar to those of *EGR1*^+^ monocytes (Fig. 4c, f and Supplementary Fig. S9d). *MX1*^+^ LDNs were superior IFN-responsive than other subsets (Fig. 4d, f). *CEACAM8*^+^ LDNs exhibited outstanding activities in innate immune defenses, including producing antimicrobial peptides, neutrophil degranulation, as well as in tissue remodeling such as extracellular matrix formation, by the high expression of antimicrobial and oxidant-scavenging genes such as *PGLYRP1*, *S100A* family members, *BPI*, *LCN2* and *LTF*, *etc*. (Fig. 4e, f). Collectively, the different immune profiles implied that *MME*^hi^, *MX1*^+^ and *PI3*^+^ LDNs were functional at cytokine-responsive and production, while *CEACAM8*^+^ LDNs were biased to direct immune defense.

Another significant difference between the subsets of LDNs lay in the metabolic pathways, in which nicotinamide salvaging was the metabolic feature of *MME*^hi^, *MX1*^+^ and *PI3*^+^ LDNs, while glycogenolysis process, mTOR and pentose pathways were the main metabolic features of the *CEACAM8*^+^ LDNs (Fig. 4f and Supplementary Fig. S9e), further indicating that differences in immune functions and processes were closely associated with metabolic pathways. Additionally, these LDN clusters were also present in COVID-19 convalescents infected by the ancestral strain based on scRNA-seq datasets, and the enrichment signaling pathways were analogical (Supplementary Fig. S10a–c). In summary, mild to moderate convalescents exhibited differences in responsive LDNs, with IL-1β- or IFN-responsiveness or selectively expressing hallmark *PI3*.

### Polarization of *EGR1*^hi^ monocytes to *CCL3*^hi^ monocytes in Omicron convalescents

**Fig. 5:**
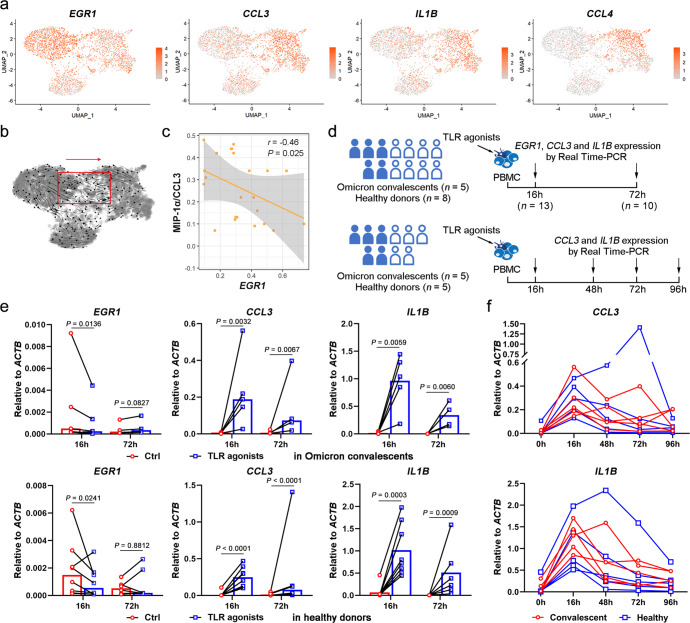
The infection signal-polarized differentiation of *EGR1*^hi^ monocytes to *CCL3*^hi^ mature monocytes in Omicron convalescents. **a** UMAP plots showing the expression of *CCL3*, *EGR1*, *IL1B* and *CCL4* in the *EGR1*^+^ monocytes by WTA scRNA-seq. **b** RNA velocity analysis of *EGR1*^+^ monocytes showing that *EGR1*^hi^ monocytes may differentiate to *CCL3*^hi^ monocytes. **c** Association between the *EGR1* expression by TTA scRNA-seq and plasma levels of MIP-1α/CCL3 in convalescents (*n* = 7 for mild and *n* = 16 for moderate). **d**–**f** The TLR agonists-induced expression of *EGR1*, *CCL3* and *IL1B* in PBMCs of the Omicron convalescents and healthy donors. The experimental flow chart is shown in (**d**). PBMCs derived from Omicron convalescents and healthy donors were stimulated with LPS (100 ng/ml) plus PolyIC (25 ųg/ml) for indicated time and the expression of *EGR1*, *CCL3* and *IL1B* were determined by Real-time PCR assays. Relative expression to *ACTB* was shown. Samples at 16 h included five convalescents and eight healthy donors, while at 72 h included four convalescents and six healthy donors due to limitations in the amount of PBMC isolated from samples. The relative expression of *EGR1*, *CCL3* and *IL1B* in PBMCs of the Omicron convalescents is shown in the upper panel of (**e**) and that of healthy donors is shown in the lower panel of (**e**). Ratio paired *t*-test. Samples from five convalescents and five healthy donors were used in dynamic curve and shown in (**f**)

EGR1 is one of the transcription factors required for monopoiesis from common myeloid progenitors.^29^ We therefore speculated that the *EGR1*^+^ monocytes which predominated in healthy donors might be the precursors of other responsive monocytes. Additionally, our previous study has demonstrated the presence of pro-inflammatory *CCL3*^+^ monocytes in severe acute COVID-19.^30^ Here, we found that a few *CCL3*^+^ monocytes were found in convalescents, while the *EGR1*^+^ monocytes in healthy individuals expressed high levels of *CCL3* transcripts. To ascertain the issue, we focused on the CD14^+^*EGR1*^+^ monocytes and reanalyzed them using WTA scRNA-seq data of healthy samples. We found that the CD14^+^*EGR1*^+^ monocytes had three subclusters, of which two subclusters were discriminated by the expression of inflammatory cytokines and chemokines such as *CCL3*, *CCL4* and *IL1B* and that of *EGR1* (Fig. 5a). The results implied that *EGR1*^hi^*CCL3*^lo^ monocytes might be naive monocytes newly differentiated from monopoiesis while those *EGR1*^lo^*CCL3*^hi^ monocytes might be derived from *EGR1*^hi^*CCL3*^lo^ monocytes.

To explicate the differentiation relationship of the *EGR1*^hi^*CCL3*^lo^ and *EGR1*^lo^*CCL3*^hi^ subsets, we performed RNA velocity analysis.^31^ As expected, the *EGR1*^hi^*CCL3*^lo^ cluster had the trend of differentiation to the *EGR1*^lo^*CCL3*^hi^ cluster (Fig. 5b). The results implied that down-regulation of *EGR1* expression may mark the differentiation of *EGR1*^hi^*CCL3*^lo^monocytes into pro-inflammatory *EGR1*^hi^*CCL3*^hi^ monocytes upon stimulation. To further verify the hypothesis, we performed correlation analysis between the *EGR1* expression in monocytes and plasma levels of cytokines and chemokines in convalescents, and found that the *EGR1* expression was significantly reversely correlated with the plasma CCL3 level (Fig. 5c).

Based on in vitro experiments, we stimulated PBMCs with TLR agonists for distinct periods, and found that the expression of *EGR1* was downregulated whereas those of *CCL3* and *IL1B* were upregulated in the acute phase, but with the stimulating progression, the levels of *CCL3* and *IL1B* gradually downregulated to the levels similar as to those of control (Fig. 5d–f).

Recruitment of *EGR1*^+^ monocytes from bone marrow to peripheral blood and transition to *CCL3*^+^ monocytes were also observed in scRNA-seq data on PBMCs from healthy donors, hospitalized patients with severe influenza, asymptomatic, mild or severe COVID-19 in the acute phase^32^ (Supplementary Fig. S11a–e), suggesting the wide presence of bone marrow myeloid recruitment and transition to *CCL3*^+^ monocytes after infection.

### Post-acute sequelae of COVID-19 mediated by IFN-responsive immune cells and cytokines

In clinical practice and rehabilitation management, we found that some COVID-19 patients continued to suffer from acute phase symptoms or new symptoms emerged after negative PCR test for SARS-CoV-2, termed post-acute sequelae of COVID-19 (PASC). The PASC of the convalescents in this study includes (1) General symptoms: fatigue; (2) Respiratory symptoms: cough, nasal congestion, nasal discharge, sore throat; (3) Digestive symptoms: diarrhoea; (4) Neurological symptoms: allotriogeusia, heterosmia; (5) Skin and mucous membrane symptoms: rash, conjunctivitis, mucosal inflammation; and (6) Circulation symptoms: hypotension, summarized in Supplementary Table. S5–S7.

**Fig. 6:**
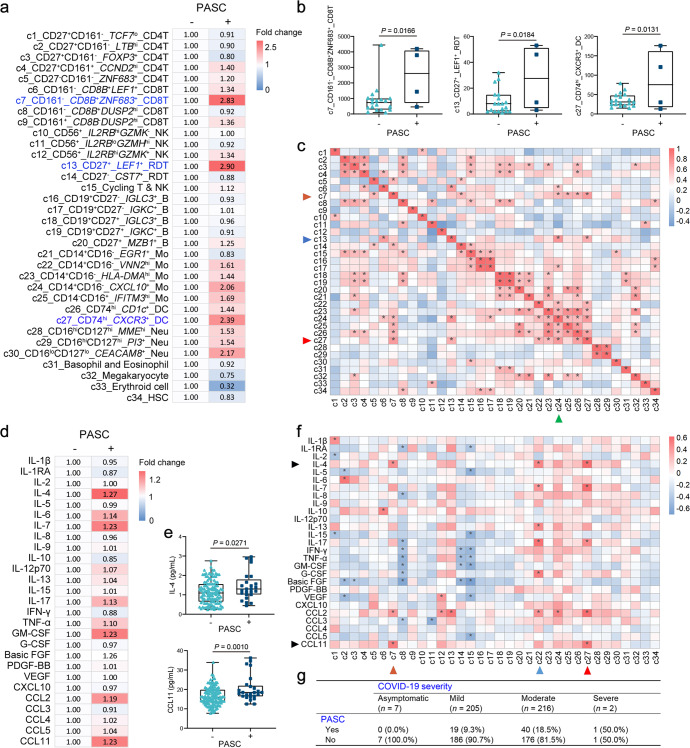
Association of post-acute sequelae of COVID-19 (PASC) and IFN-responsive immune cells and cytokines/chemokines. **a** The fold change of the cell number in each cluster between Omicron convalescents with PASC (*n* = 4) or without PASC (*n* = 19). **b** Box plots showing the differences in cell number between Omicron convalescents with or without PASC. *t*-test. **c** Heatmap showing correlations across each cluster in Omicron convalescents. *Spearman* rank correlation test. **d** The fold change of plasma concentrations of cytokines and chemokines between Omicron convalescents with PASC (*n* = 25) or without PASC (*n* = 118). **e** Box plots showing the differences in plasma concentrations of cytokines and chemokines between Omicron convalescents with or without PASC. *t*-test. **f** Heatmap showing correlations across cytokines/chemokines and cell clusters in Omicron convalescents. *Spearman* rank correlation test. **g** The correlation between PASC and acute COVID-19 severity in all infected individuals of the Omicron epidemic in Tianjin during January–February 2022 (*n* = 430)

To explicate the immune profile of post-COVID conditions, we first compared the cell numbers of each cluster in Omicron convalescents with or without PASC. We found that *ZNF683*^+^ CD8 T cells, *LEF1*^+^ T_γδ_ cells and *CXCR3*^+^ DCs were significantly increased in the peripheral blood of Omicron convalescents with PASC (Fig. 6a, b). The three cell clusters, in addition to correlating with each other, were robust and positively correlated with CD14^+^*CXCL10*^+^ monocytes (Fig. 6c and Supplementary Fig. S12a–c), suggesting that PASC may be modulated by monocytes with IFN-responsiveness. Additionally, CCL11 and IL-4 were extraordinarily increased in plasma of patients with PASC (Fig. 6d, e), associated with *ZNF683*^+^ CD8 T cells, *VNN2*^hi^ monocytes and *CXCR3*^+^ DCs (Fig. 6f and Supplementary Fig. S12d). Previous studies have illustrated that the expression of CCL11 is regulated by interferons, while IL-4 has a significant antagonistic effect on interferons.^33–35^ These results implied that IFN-responsive cytokines were important regulators of PASC.

Furthermore, we investigated the probability of PASC in patients with different acute COVID-19 severity in all infected individuals of the Omicron epidemic in Tianjin during January-February 2022. Because of the limited number of asymptomatic and severely infected individuals, we mainly compared the incidence of PASC in mild and moderate convalescents. The moderate COVID-19 patients who had experienced pneumonia in the acute phase were more likely to have post-COVID conditions than those who had experienced only mild symptoms (18.5% in moderate and 9.3% in mild, *P* = 0.006, Fig. 6g). It is consistent with the finding that IFN-responsive monocytes were exceedingly increased in moderate convalescents. In summary, the emergence and persistence of PASC may be attributed to IFN-responsive monocytes and cytokines.

## Discussion

Much work has delineated the immune features of wild-type or early variants of SARS-CoV-2 infection, but the immune imprinting profiles of innate immunity in the Omicron variant infection remain unclear. Here, we provided detailed characterization of the innate immunity of individuals recovering from Omicron infection. Further, we found that monocytes and LDNs of COVID-19 convalescents recovered from Omicron infection were imprinted with IL-1β- or IFN- responsive signatures. Additionally, the emergence of massive *CCL3*^hi^ monocytes in circulation has been reported by many previous studies from severe COVID-19 patients in the acute phase,^36,37^ our finding that *EGR1*^hi^ monocytes can transit to *CCL3*^hi^ monocytes implied the potential sources of *CCL3*^hi^ monocytes in severe COVID-19 patients.

*PI3*^+^ LDNs and *VNN2*^hi^ monocytes might be critical for Omicron patients with only asymptomatic or mild symptoms. We identified for the first time a protective subset of LDNs with high expression of *PI3*. PI3 is a serine proteinase inhibitor targeting proteinase 3 that participates in tissue injury.^38^ PI3 has also been reported to reduce the amounts of active IL-1β from extracellular pro-IL-1β cleaved by proteinase 3.^39^ Single nucleotide polymorphisms (SNPs) in the *PI3* gene or low PI3 plasma levels show an association with the increased risk of ARDS,^27,40^ suggesting that PI3 can play a protective role in controlling the progression of COVID-19. *VNN2*^hi^ monocytes may be another subset affecting the disease severity. *VNN2*^hi^ monocytes are monocytic myeloid-derived suppressive cells that inhibit T cell responses, present in healthy individuals as an immune-homeostasis compartment to an appropriate immune response as well as massively increased in some tumors for building an immune inhibitory milieu.^41,42^ The increased *VNN2*^hi^ monocytes should be one important mechanism to reduce the immune injury from T cells in the setting of Omicron infection, and possibly responsible for the timely quelling the antiviral T cell response in mild convalescents. The differentiation of *VVN2*^hi^ monocytes largely depends on G-CSF and IL-1β. IL-1β, despite a potent inflammatory cytokine, is confirmed to be involved in the differentiation of myeloid-derived suppressive cells.^43–45^ We are still uncertain on the mechanisms underlying the differentiation of *PI3*^+^ LDNs, which need to be studied further. Protective *PI3*^+^ LDNs and *VVN2*^hi^ monocytes allow a fine balance between immune responses and regulation, possibly critical for the Omicron variant causing only asymptomatic to mild clinical symptoms.

Previous studies have reported severe humoral immune imbalances and cytokine storms in severe COVID-19 as well as the important role of monoclonal antibody therapy targeting certain cytokines.^46,47^ Here, we observed the differentiation of naive *EGR1*^+^ monocytes into *CCL3*^+^ monocytes at the early stage of bone marrow myeloid recruitment after Omicron infection and found the same transition in influenza patients, elucidating that *EGR1*^+^ monocytes may be the source of the cytokine storm following viral infections. Additionally, Omicron infection exhibits a delicate balance among cytokines and chemokines. Our study reveals the association between cytokine preferences and disease severity. IL-9 and IL-1β are related to the convalescents experienced mild symptoms, while IFN pathway products such as CXCL10 prefer the moderate convalescents. As a biomarker of cytokine storms, IL-1β is proportional to the severity of disease in viral infections such as SARS-CoV-2 ancestral strain and MERS-CoV.^48–50^ However, we found that IL-1β levels were higher in mild convalescents than in those with moderate disease, and this Omicron-specific pattern may be associated with non-severe clinical symptoms. Insufficient or delayed IFN response, later than the peak of clinical symptoms and viral replication, is a prominent feature of the acute phase of severe COVID-19.^5,51,52^ Additionally, the IFN response decreased over time during the inflammation resolution. Our data indicate that the moderate convalescents may have a delayed IFN response than mild ones during the acute infection phase, which should be validated by further investigation. On the other side of the balance, protective cytokines may also be responsible for attenuating serious COVID-19. IL-9, secreted by the group 2 innate lymphocytes or T helper 9 cells, promotes cupped cell proliferation and mucus production, thereby facilitating tissue repair of epithelial injury during lung inflammation.^53–56^ However, IL-9 was undetected in all the clusters of PBMCs, and the sources of IL-9 still needed to be clarified.

The COVID-19 PASC has gained extensive attention due to the affliction on more individuals. Several studies suggested that chronic inflammation is a potential cause of PASC.^57,58^ The classic symptoms of chronic inflammation, such as fatigue, fever and pain, overlap highly with those of the post-acute sequelae of non-persistent viral infections, and patients with Long COVID typically have high levels of pro-inflammatory cytokines.^21,59^ Dysregulation of the innate immune system is certainly an important contributing factor to chronic inflammation. Previous studies have reported that convalescents developed long-term immune system disturbances even after the acute infection period,^60–62^ which is consistent with our observations. Elevated frequencies of responsive T cells and some innate cells and levels of CCL11 and IL-4 in convalescents with PASC confirm a direct correlation between persistent immune responses and PASC. The bias of IFN-responsive signatures in convalescents with PACS indicates that PACS may be the consequences of IFN-associated inflammation and/or secondary organ and tissue injuries possibly caused by the persistent effector/memory T cells. Overall, we provide a piece of the puzzle at single-cell resolution to better understand the immune spectrum of convalescents who are experiencing PASC.

There are several limitations of this study. The data for the post-COVID conditions were collected 4-8 weeks after onset when the patients were SARS-CoV-2 negative and had no evidence of reinfection. Long-term observation of PASC in convalescents is still needed to explore the immune spectrum of Long COVID. Second, the number of samples from individuals without vaccine immunization was limited, limiting the potential to reveal the full spectrum of the impacts on innate immunity by vaccination. This study was performed in the first wave of SARS-CoV-2 Omicron epidemic caused by the BA.1 sublineage. Therefore, the impact of the new sublineages of Omicron on the immune system still needs further investigation and research.

In summary, our study provides important findings with the perspective of innate immunity imprints for understanding the Omicron variant infection severity and post-acute sequelae of COVID-19.

## Materials and methods

### Patients and healthy donors

In this study, we recruited 143 SARS-CoV-2 Omicron BA.1-infected individuals (confirmed by RT-PCR test or second-generation sequencing) in Tianjin First Central Hospital and 48 healthy individuals with RT-PCR test negative for SARS-CoV-2 and no virus-specific serum IgM and IgG in Shanghai Changhai Hospital. COVID-19 patients were from the SARS-CoV-2 pandemic caused by Omicron BA.1 between January-February 2022 in Tianjin, as described in our previous study.^8^ According to WHO living guidance for clinical management of COVID-19,^63^ mild COVID-19 patients were defined as having mild clinical symptoms (e.g. fever, cough, etc.) without imaging evidence of viral pneumonia or hypoxia, while moderate cases had CT-certified manifestations of pneumonia, but SpO_2_ ≥ 90% on room air. Those with active viral hepatitis, active tuberculosis, abnormal immune function, on immunosuppressive status, antibody, or antiviral therapy were excluded. The SARS-CoV-2 vaccination status and clinical information on COVID-19 disease were recorded, and then peripheral blood was collected. The median interval between the infection and sampling was 42 days (interquartile, 41–44), and the median interval between the last vaccination and sampling in patients was 115 days (82–232). While the interval from last vaccination to sampling in healthy individuals was 46 days (41–48). Relevant experiments regarding Omicron BA.1 convalescent individuals were approved by the Ethics Committee of Tianjin First Central Hospital, Nankai University (No. 2022N045KY). Relevant experiments regarding vaccinated individuals were approved by the Ethics Committee of the First Affiliated Hospital of Guangzhou Medical University (2021-78) and the Ethics Committee of Naval Medical University. All patients and healthy donors had signed written informed consent forms.

### Isolation of PBMCs and plasma

Peripheral blood was first diluted 1:1 with phosphate-buffered saline (PBS) (Invitrogen) containing 2% fetal bovine serum (FBS) (Gibco), followed by Ficoll (Cytiva) gradient centrifugation. Plasma was separated at this step and stored at −80 °C for further storage. After erythrocyte lysis and washing, fresh isolated PBMCs are directly processed for single-cell sequencing or resuspended in FBS containing 10% dimethyl sulfoxide (Sigma–Aldrich) at −80 °C for further preservation.

### Culture of PBMCs

Fresh or thawed PBMCs at 5 × 10^5^/ml were stimulated with TLR agonists (LPS: 100 ng/ml, PolyIC:25 μg/ml) for the indicated time, and then the expression of *IL1B*, *CCL3* and *EGR1* at mRNA levels was detected using real-time PCR.

### Measurement of cytokines and chemokines

To evaluate the immune characteristics of Omicron BA.1 infection convalescents, the cytokine and chemokine concentrations in the plasma of convalescents and matched healthy donors were measured by the Bio‐Plex pro human cytokine assays (27‐Plex #12007283; Bio‐Rad) and Luminex 200 system,^64^ including interleukins (IL‐1β, IL‐1RA, IL‐2, IL‐4, IL‐5, IL‐6, IL‐7, IL‐8, IL‐9, IL‐10, IL‐12 (p70), IL‐13, IL‐15, and IL‐17), interferons (IFN‐γ), tumor necrosis factor (TNF‐ɑ), colony‐stimulating factors (GM‐CSF, G‐CSF), growth factors (basic fibroblast growth factor [FGF], platelet‐derived growth factor [PDGF‐BB], and vascular endothelial growth factor [VEGF]), and chemokines (inducible protein‐10 [IP‐10]/CXCL10, monocyte chemoattractant protein‐1 [MCP‐1]/CCL2, macrophage‐inflammatory protein [MIP‐1ɑ]/CCL3, MIP‐1β/CCL4, regulated upon activation normal T‐cell expressed and secreted [RANTES]/CCL5, and Eotaxin/CCL11).

### Real-time PCR

Total RNA was extracted with TRIzol reagent and reverse transcribed, and the resulting cDNA was quantified by real-time PCR assays using SYBR Premix ExTaq kit on a LightCycler (Roche, Basel). Data of each sample were normalized to the expression of *ACTB*. PCR primers are listed as the following: *CCL3*, Forward, 5ʹ-AGTTCTCTGCATCACTTGCTG-3ʹ; Reverse, 5ʹ-CGGCTTCGCTTGGTTAGGAA-3ʹ; *IL1B*, Forward, 5ʹ-ATGATGGCTTATTACAGTGGCAA-3ʹ; Reverse, 5ʹ-GTCGGAGATTCGTAGCTGGA-3ʹ; *EGR1*, Forward, 5ʹ-GGTCAGTGGCCTAGTGAGC-3ʹ; Reverse, 5ʹ-GTGCCGCTGAGTAAATGGGA-3ʹ.

### Single-cell RNA sequencing and surface proteome profiling

PBMCs from 23 Omicron BA.1 convalescents with different vaccinations, who experienced mild or moderate COVID-19, and 6 age- and gender- matched healthy controls patients were processed for single-cell sequencing. Specifically, the cells were stained with a specific sample Tag (BD Rhapsody Human single-cell multiplexing kit) and 30 AbSeq antibodies against major human immune markers (BD Abseq immune discovery panel, Supplementary Table. S8) for 30 min on ice. After extensively washing, equal amounts of cells with different sample tags were pooled together and a max of 60,000 cells was loaded on a BD Rhapsody Cartridge. Single-cell capture and cDNA library preparation were performed using the BD Rhapsody Express Single-Cell Analysis System (BD Biosciences) according to the manufacturer’s instructions. Libraries of single-cell transcriptomes targeting immune profiles (TTA) and whole transcriptomes (WTA), Ab tagged index sequences targeting 30 immune cell-associated surface antigens and multiple sample tags were prepared using the BD Rhapsody TTA and WTA amplification kit according to the manufacturer’s instructions. BD Rhapsody™ immune response panel was used to prepare the mRNA targeted library which contains primer pairs that target 397 genes commonly expressed in human immune cells. Sequencing was performed on Illumina HiSeq 6000 platform (Novogen). The FASTQ files of sequencing were analyzed using BD Rhapsody Analysis Pipeline v1.10.1 to obtain an expression matrix.^65^

### Statistical analysis

The computational analysis of single-cell RNA sequencing data sets is mainly performed using the R package Seurat (version 4.1).^66^ To remove the effect of double cells, we used the DoubletFinder package (version 2.0.3) to identify and removed suspicious double cells.^67^ Cells in which <50 targeted mRNA were deleted. The expression matrix was merged by Seurat function merge, and normalization was performed by function SCTransform.^68^ PCA was performed with RunPCA function using all genes. The top 30 PCs were used as inputs to utilize a shared nearest neighbor graph constructed by FindNeighbors function. We used FindCluster function to identify cell clusters with the resolution parameter set to 0.15. To further identify the clusters, we divided all cells into 4 major clusters: monocytes & DCs, T cells, B cells, LDNs with other cells. For each of the 4 major clusters, we performed the pipeline again as described above. In the end, all obtained clusters were displayed on the overall UMAP graph.

For WTA analysis, because the RNA sequenced by TTA and WTA were from the same cell, we can link TTA data and WTA data by the UMI of beads, and those cells only detected at WTA were removed. Before the analysis of WTA, we selected the beads which were identified in monocytes in TTA. We used the function SCTransform to normalize data. Then, we abandoned cells that were not identified as LDNs by WTA. In the end, with the PCs of 20 and the resolution of 0.1, we splited the LDNs into 4 independent clusters. Monocytes were treated in the same way as LDNs.

In order to identify the marker genes of a specific cluster, the Seurat FindAllMarkers function was applied, which was limited to the genes detected in more than 25% of the cells, and the average fold change difference is 0.25 or more. We calculated the markers of protein and RNA in each group separately.

To identify the biological process and signaling pathways enriched by each cluster of monocytes and LDNs, we downsampled each cluster to 1000 cells and calculated AUC scores of pathways for each cell using the R package irGSEA (version 1.1.2).^69^ The gene sets of pathways were from Molecular Signatures Database (MSigDB, http://www.broadinstitute.org/gsea/msigdb/) (version 7.5.1).^70^ The scores of interested pathway were calculated by the Seurat AddModuleScore function. RNA velocity analysis was performed by scVelo packages (version 0.2.4).^71^

The correlation analysis between cytokines and genes was performed by using Spearman correlation. The value of cytokines was measured in the plasma of convalescents and the value of genes was the average expression of monocytes in WTA.

For cytokines and chemokines measurement, statistical significance was determined by a two-tailed Mann–Whitney U test, and *P* < 0.05 was considered statistically significant.

## Supplementary information

Supplementary Materials

## Data Availability

The single-cell RNA-seq data generated in this study have been deposited in the Genome Sequence Archive (GSA) under the accession number HRA002716. Additional single-cell RNA-seq data used in the study has been described in the text and figure legends.
