# Supplementary material for: Innate immune imprints in SARS-CoV-2 Omicron variant infection convalescents
Source: Signal Transduct Target Ther. 2022 Nov 16;7:377. doi: 10.1038/s41392-022-01237-y (PMC9666472; doi:10.1038/s41392-022-01237-y)
Supplement: Supplementary file 1 — Supplementary Materials [file 41392_2022_1237_MOESM1_ESM.docx]

Supplementary Materials for

**Innate immune imprints in SARS-CoV-2 Omicron variant infection convalescents**

Zhiqing Li^1,9^, Xiaosu Chen^2,9^, Junyan Dan^1,9^, Tianju Hu^3,9^, Ye Hu^2,9^, Shuxun Liu^1,9,*^, Yangyang Chai^3^, Yansong Shi^2^, Jian Wu^1^, Hailai Ni^4^, Jiaqi Zhu^5^, Yanfeng Wu^1^, Nan Li^1^, Yizhi Yu^1^, Zhongfang Wang^6^, Jincun Zhao^6^, Nanshan Zhong^6^, Xianwen Ren^7,*^, Zhongyang Shen^8,*^ and Xuetao Cao^1,2,3,*^

Correspondence to: Shuxun Liu (liusx@immunol.org), Xianwen Ren (renxwise@cpl.ac.cn), Zhongyang Shen (zhongyangshen@nankai.edu.c), or Xuetao Cao (caoxt@immunol.org)

**This PDF file includes:**

Materials and Methods

Figures. S1 to S12

Tables S1 to S8

References

Materials and Methods

Patients and healthy donors

In this study, we recruited 143 SARS-CoV-2 Omicron BA.1-infected individuals (confirmed by RT-PCR test or second-generation sequencing) in Tianjin First Central Hospital and 48 healthy individuals with RT-PCR test negative for SARS-CoV-2 and no virus-specific serum IgM and IgG in Shanghai Changhai Hospital. COVID-19 patients were from the SARS-CoV-2 pandemic caused by Omicron BA.1 between January-February 2022 in Tianjin, as described in our previous.[^1^](#_ENREF_1) According to WHO living guidance for clinical management of COVID-19,[^2^](#_ENREF_2) mild COVID-19 patients were defined as having mild clinical symptoms (e.g. fever, cough, etc.) without imaging evidence of viral pneumonia or hypoxia, while moderate cases had CT-certified manifestations of pneumonia, but SpO2 ≥ 90% on room air. Those with active viral hepatitis, active tuberculosis, abnormal immune function, on immunosuppressive status, antibody, or antiviral therapy were excluded. The SARS-CoV-2 vaccination status and clinical information on COVID-19 disease were recorded, and then peripheral blood was collected. The median interval between the infection and sampling was 42 days (interquartile, 41-44), and the median interval between the last vaccination and sampling in patients was 115 days (82-232). While the interval from last vaccination to sampling in healthy individuals was 46 days (41-48). Relevant experiments regarding Omicron BA.1 convalescent individuals were approved by the Ethics Committee of Tianjin First Central Hospital, Nankai University (No. 2022N045KY). Relevant experiments regarding vaccinated individuals were approved by the Ethics Committee of the First Affiliated Hospital of Guangzhou Medical University (2021-78) and the Ethics Committee of Naval Medical University. All patients and healthy donors had signed written informed consent forms.

Isolation of PBMCs and plasma

Peripheral blood was first diluted 1:1 with phosphate-buffered saline (PBS) (Invitrogen) containing 2% fetal bovine serum (FBS) (Gibco), followed by Ficoll (Cytiva) gradient centrifugation. Plasma was separated at this step and stored at -80℃ for further storage. After erythrocyte lysis and washing, fresh isolated PBMCs are directly processed for single-cell sequencing or resuspended in FBS containing 10% dimethyl sulfoxide (Sigma-Aldrich) at -80 ℃ for further preservation.

Culture of PBMCs

Fresh or thawed PBMCs at 5×105/ml were stimulated with TLR agonists (LPS: 100ng/ml, PolyIC:25mg/ml) for the indicated time, and then the expression of IL1B, CCL3 and EGR1 at mRNA levels was detected using real-time PCR.

Measurement of cytokines and chemokines

To evaluate the immune characteristics of Omicron BA.1 infection convalescents, the cytokine and chemokine concentrations in the plasma of convalescents and matched healthy donors were measured by the Bio‐Plex pro human cytokine assays (27‐Plex #12007283; Bio‐Rad) and Luminex 200 system,[^3^](#_ENREF_3) including interleukins (IL‐1β, IL‐1RA, IL‐2, IL‐4, IL‐5, IL‐6, IL‐7, IL‐8, IL‐9, IL‐10, IL‐12 (p70), IL‐13, IL‐15, and IL‐17), interferons (IFN‐γ), tumor necrosis factor (TNF‐ɑ), colony‐stimulating factors (GM‐CSF, G‐CSF), growth factors (basic fibroblast growth factor [FGF], platelet‐derived growth factor [PDGF‐BB], and vascular endothelial growth factor [VEGF]), and chemokines (inducible protein‐10 [IP‐10]/CXCL10, monocyte chemoattractant protein‐1 [MCP‐1]/CCL2, macrophage‐inflammatory protein [MIP‐1ɑ]/CCL3, MIP‐1β/CCL4, regulated upon activation normal T‐cell expressed and secreted [RANTES]/CCL5, and Eotaxin/CCL11).

Real-time PCR

Total RNA was extracted with TRIzol reagent and reverse transcribed, and the resulting cDNA was quantified by real-time PCR assays using SYBR Premix ExTaq kit on a LightCycler (Roche, Basel). Data of each sample were normalized to the expression of *ACTB*. PCR primers are listed as the following: CCL3, Forward, 5’-AGTTCTCTGCATCACTTGCTG-3’; Reverse, 5’-CGGCTTCGCTTGGTTAGGAA-3’; IL1B, Forward, 5’-ATGATGGCTTATTACAGTGGCAA-3’; Reverse, 5’-GTCGGAGATTCGTAGCTGGA-3’; EGR1, Forward, 5’-GGTCAGTGGCCTAGTGAGC-3’; Reverse, 5’-GTGCCGCTGAGTAAATGGGA-3’.

Single-cell RNA sequencing and surface proteome profiling

PBMCs from 23 Omicron BA.1 convalescents with different vaccinations, who experienced mild or moderate COVID-19, and 6 age- and gender- matched healthy controls patients were processed for single-cell sequencing. Specifically, the cells were stained with a specific sample Tag (BD Rhapsody Human single-cell multiplexing kit) and 30 AbSeq antibodies against major human immune markers (BD Abseq immune discovery panel, Supplementary Table S8) for 30 min on ice. After extensively washing, equal amounts of cells with different sample tags were pooled together and a max of 60000 cells was loaded on a BD Rhapsody Cartridge. Single-cell capture and cDNA library preparation were performed using the BD Rhapsody Express Single-Cell Analysis System (BD Biosciences) according to the manufacturer’s instructions. Libraries of single-cell transcriptomes targeting immune profiles (TTA) and whole transcriptomes (WTA), Ab tagged index sequences targeting 30 immune cell-associated surface antigens and multiple sample tags were prepared using the BD Rhapsody TTA and WTA amplification kit according to the manufacturer’s instructions. BD Rhapsody™ immune response panel was used to prepare the mRNA targeted library which contains primer pairs that target 397 genes commonly expressed in human immune cells. Sequencing was performed on Illumina HiSeq 6000 platform (Novogen). The FASTQ files of sequencing were analyzed using BD Rhapsody Analysis Pipeline v1.10.1 to obtain an expression matrix.[^4^](#_ENREF_4)

Statistical analysis

The computational analysis of single-cell RNA sequencing data sets is mainly performed using the R package Seurat (version 4.1).[^5^](#_ENREF_5) To remove the effect of double cells, we used the DoubletFinder package (version 2.0.3) to identify and removed suspicious double cells.[^6^](#_ENREF_6) Cells in which <50 targeted mRNA were detected. The expression matrix was merged by Seurat function merge, and batch effects were eliminated by function SCTransform.[^7^](#_ENREF_7) PCA was performed with RunPCA function using all genes. The top 30 PCs were used as inputs to utilize a shared nearest neighbor graph constructed by FindNeighbors function. We used FindCluster function to identify cell clusters with the resolution parameter set to 0.15. To further identify the clusters, we divided all cells into 4 major clusters: monocytes&DCs, T cells, B cells, low-density neutrophils (LDNs) with other cells. For each of the 4 major clusters, we performed the pipeline again like all cells. In the end, we display all obtained clusters on the overall UMAP graph.

For WTA analysis, because the RNA sequenced by TTA and WTA were from the same cell, we can link TTA data and WTA data by the UMI of beads, and those cells only detected at WTA were removed. Before the analysis of WTA, we selected the beads which were identified in monocytes in TTA. For eliminating the batch effects in WTA, we use the function SCTransform to make no overall difference in cells from different volunteers. Then, we abandoned cells identified as not monocytes by WTA. In the end, with the pcs of 20 and the resolution of 0.24, we split the monocytes into 8 independent clusters. LDNs are treated in the same way as monocytes.

In order to identify the marker genes of a specific cluster, the Seurat FindAllMarkers function is applied, which is limited to the genes detected in more than 25% of the cells, and the average fold change difference is 0.25 or more. We calculated the markers of protein and RNA in each group separately.

To identify the biological process and signaling pathways enriched by each cluster of monocytes and LDNs, we downsampled each cluster to 1000 cells and calculated AUC scores of pathways for each cell using the R package irGSEA (version 1.1.2).[^8^](#_ENREF_8) The gene sets of pathways were from Molecular Signatures Database (MSigDB, http://www.broadinstitute.org/gsea/msigdb/) (version 7.5.1).[^9^](#_ENREF_9) The scores of interested pathway were calculated by the Seurat AddModuleScore function. RNA velocity analyze was performed by scVelo packages (version 0.2.4).[^10^](#_ENREF_10)

The correlation analysis between cytokines and genes was performed by using Spearman correlation. The value of cytokines was measured in the plasma of convalescents and the value of genes was the average expression of monocytes in WTA.

For cytokines and chemokines measurement, statistical significance was determined by a two-tailed Mann–Whitney U test, and *P* < 0.05 was considered statistically significant.

Data Availability

The single-cell RNA-seq data generated in this study has been deposited in the Genome Sequence Archive (GSA) under the accession number HRA002716. Additional single-cell RNA-seq data used in the study has been described in the text and figure legends.


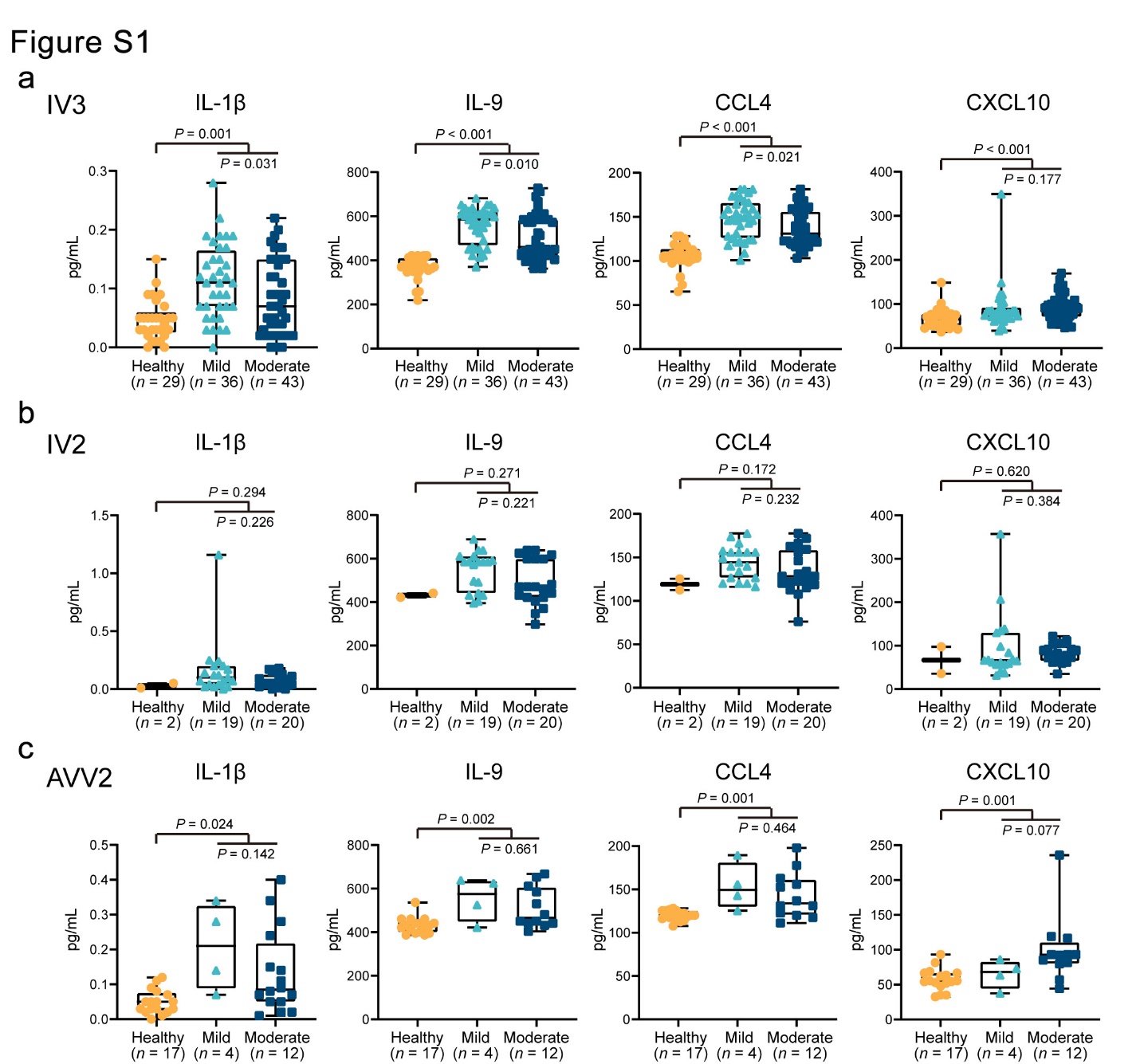


Figure S1. Cytokine quantification in convalescents and healthy individuals with different vaccination.

**a.** Cytokine quantification in convalescents (*n* = 79) and healthy individuals (*n* = 29) with 3 doses of inactivated vaccines.

**b.** Cytokine quantification in convalescents (*n* = 39) and healthy individuals (*n* = 2) with 2 doses of inactivated vaccines.

**c.** Cytokine quantification in convalescents (*n* = 16) and healthy individuals (*n* = 17) with 2 doses of adenovirus-vectored vaccines.


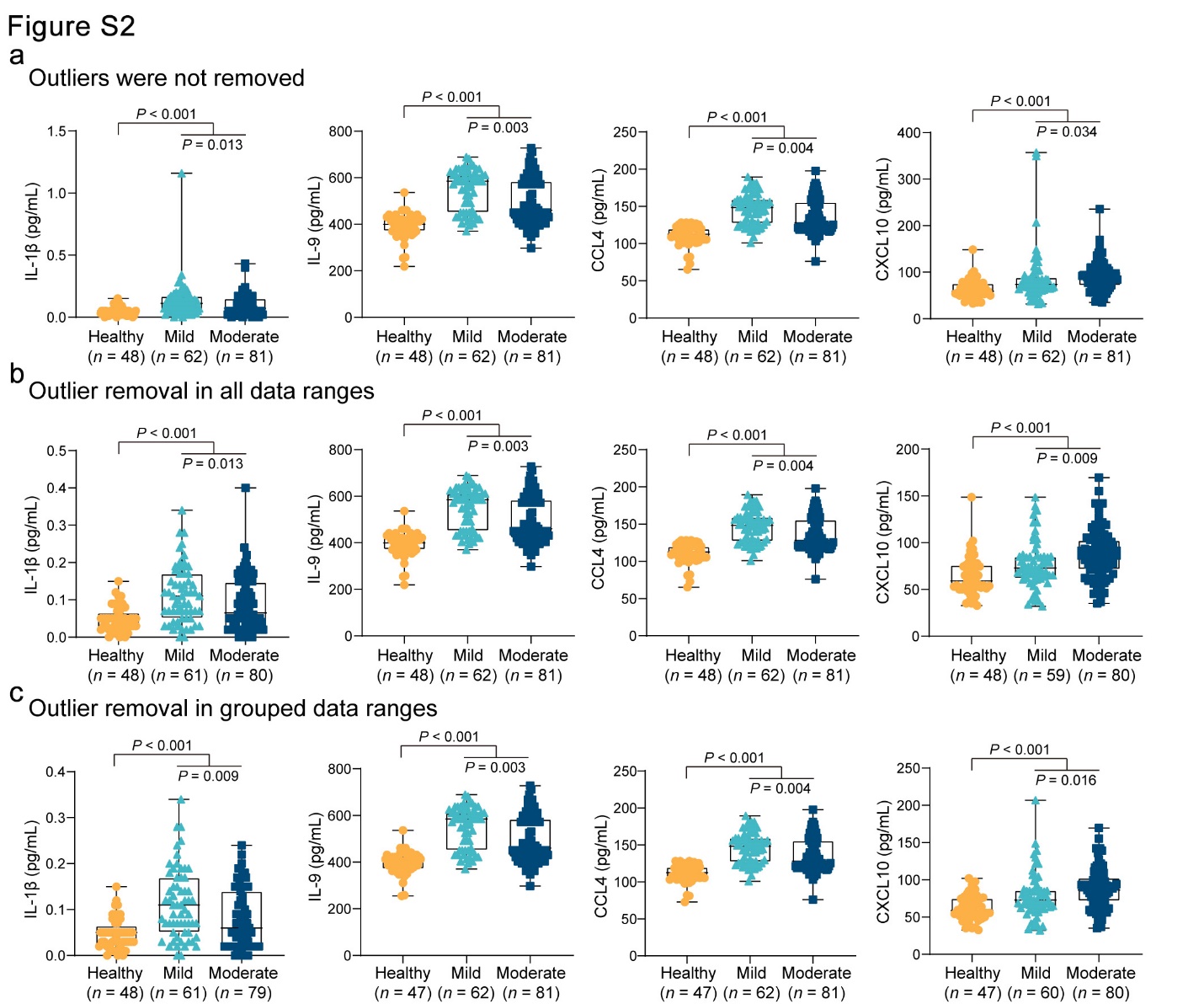


Figure S2. Cytokine quantification in Omicron convalescents and healthy individuals with the exclusion of outliers according to the PauTa criterion.

**a.** Cytokine quantification in convalescents (*n* = 62 for mild and *n* =81 for moderate) and healthy individuals (*n* = 48) without the exclusion of outliers, as shown in Fig. 1c.

**b.** Cytokine quantification in convalescents and healthy individuals after the exclusion of outliers in all date ranges.

**c.** Cytokine quantification in convalescents and healthy individuals after the exclusion of outliers in grouped data ranges.


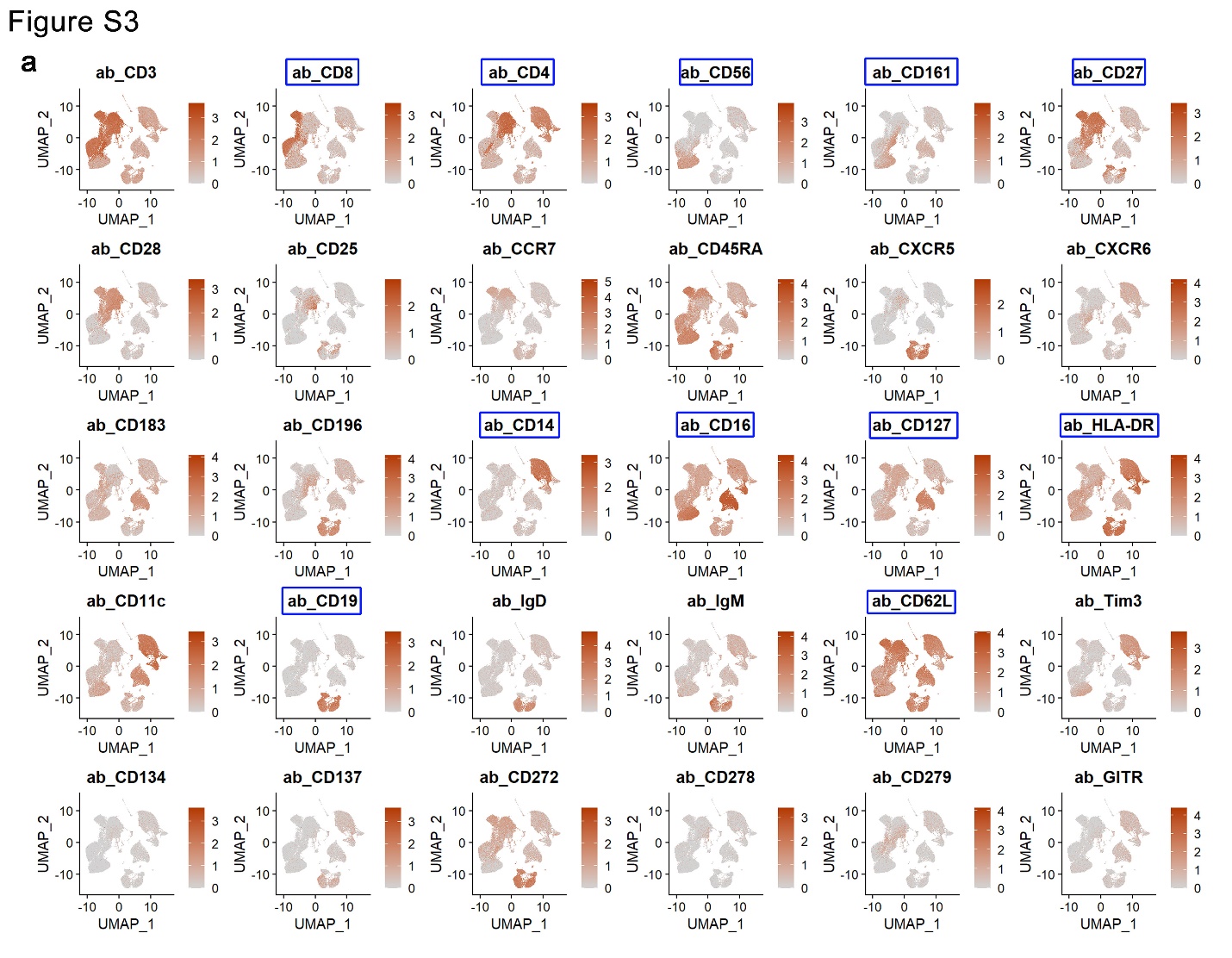


Figure S3. The expression of antigens in cell clusters from Omicron convalescents (*n* = 23) and healthy individuals (*n* = 6).

**a.** UMAP plots showing the expression of the 30 targeted surface proteins. Antigens framed in blue rectangles were used in naming the clusters.

**
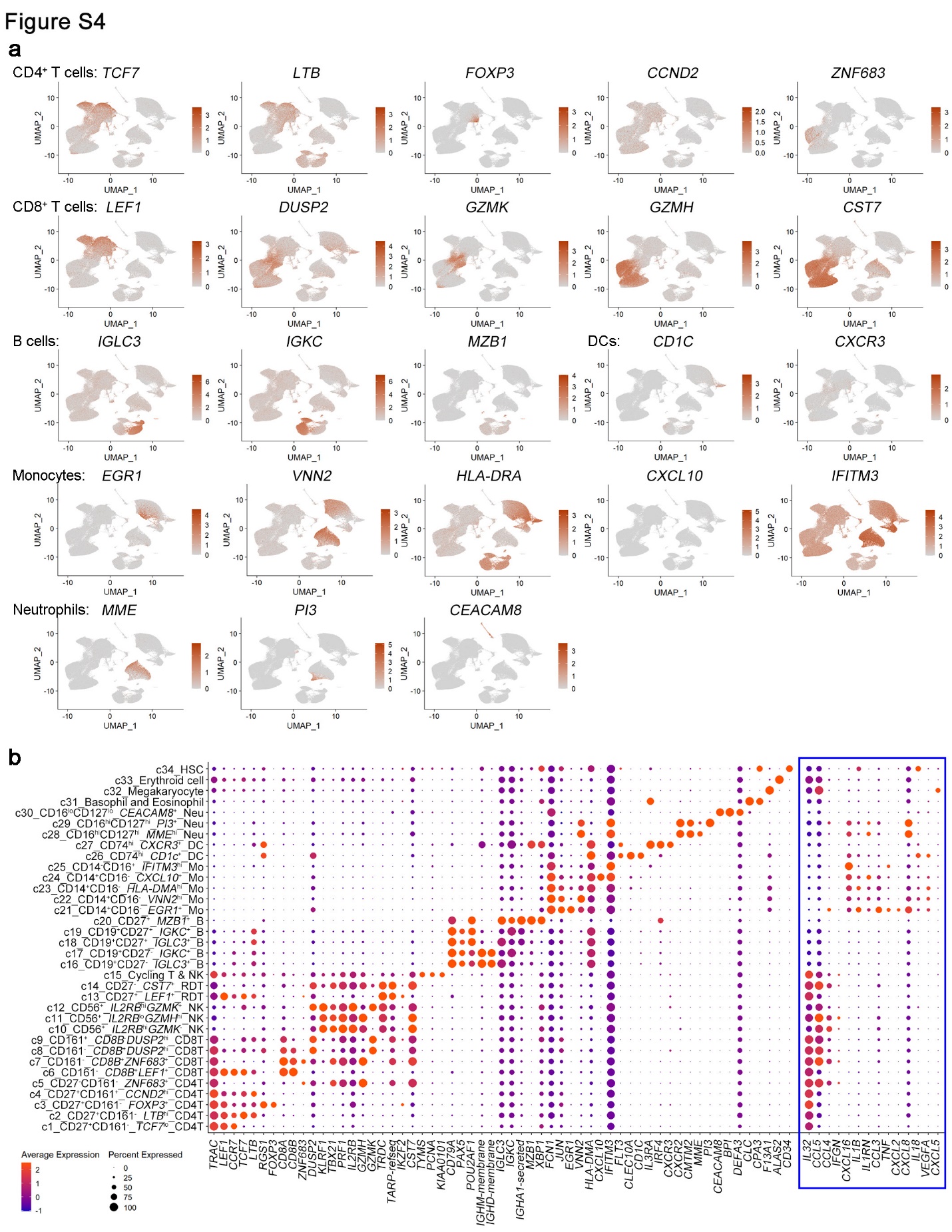
**

Figure S4. The expression of specific or abundant genes in cell clusters from Omicron convalescents (*n* = 23) and healthy individuals (*n* = 6).

**a.** UMAP plots showing the cellular distribution of marker genes with high abundance indicating cell clusters from CD4^+^ T cells (Top) and CD8^+^ T cells (the 2^nd^ panel), B cells (the 3^rd^ left panel), DCs (the 3^rd^ right panel), monocytes (the 4^th^ left panel) and LDNs (bottom) measured by TTA scRNA-seq, which were used in naming the clusters.

**b.** Dot plots showing the specific or abundant expression of genes in each cell cluster measured by TTA scRNA-seq of immune response-associated profiles. The dot plots marked by the blue rectangle showing the cellular sources of increased plasma cytokines and chemokines at transcription levels measured by TTA scRNA-seq of immune response-associated profiles.


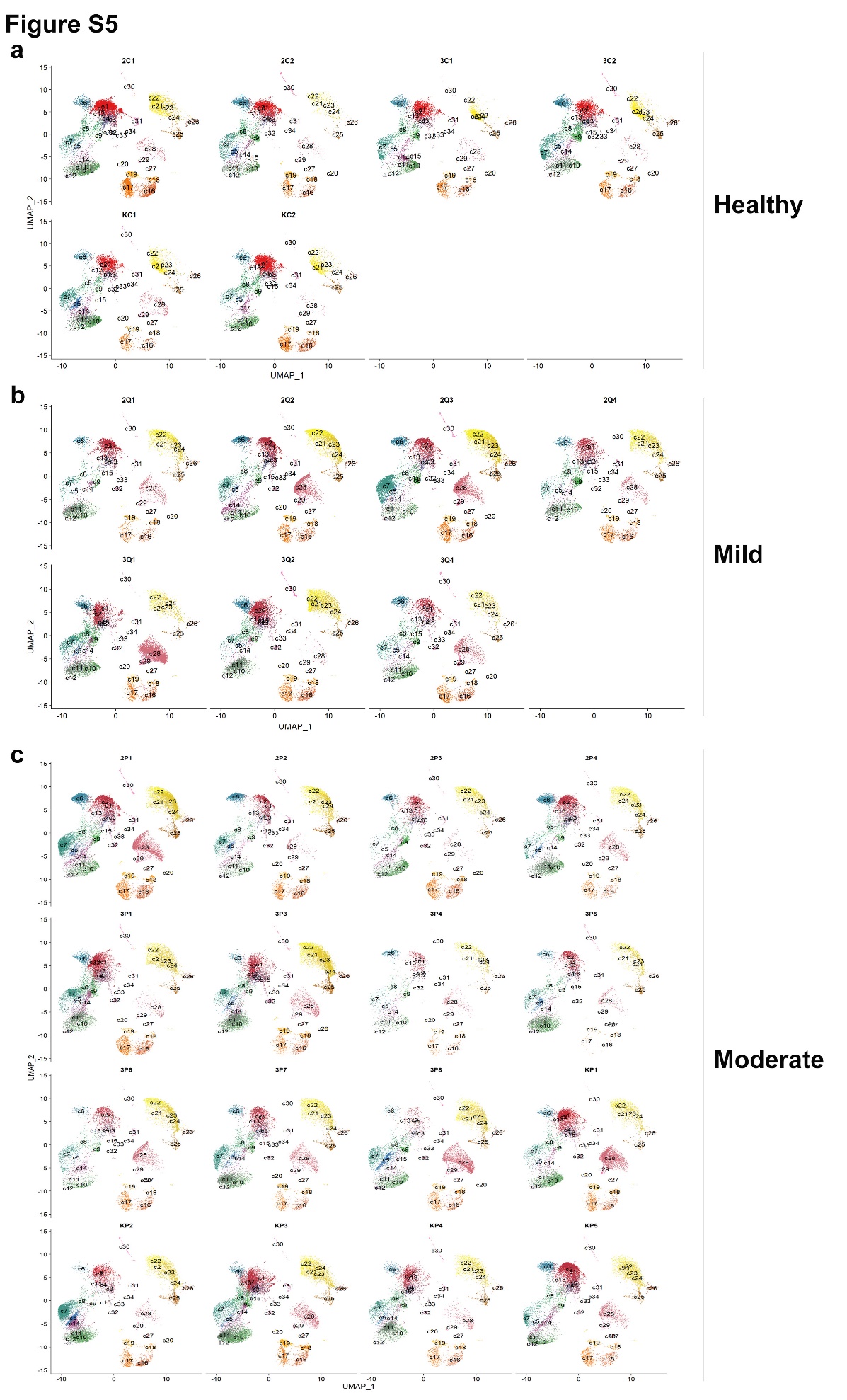


Figure S5. UMAP plots showing the cellular distribution of cell clusters in each sample measured by TTA scRNA-seq.

**a.** UMAP plots showing the cellular distribution of cell clusters in healthy samples (*n* = 6, [2C1, 2C2, 3C1, 3C2, KC1 and KC2]).

**b.** UMAP plots showing the cellular distribution of cell clusters in mild convalescents (*n* = 7, [2Q1-2Q4, 3Q1, 3Q2 and 3Q4]).

**c.** UMAP plots showing the cellular distribution of cell clusters in moderate convalescents (*n* = 16, [2P1-2P4, 3P1, 3P3-3P8 and KP1-KP5]).


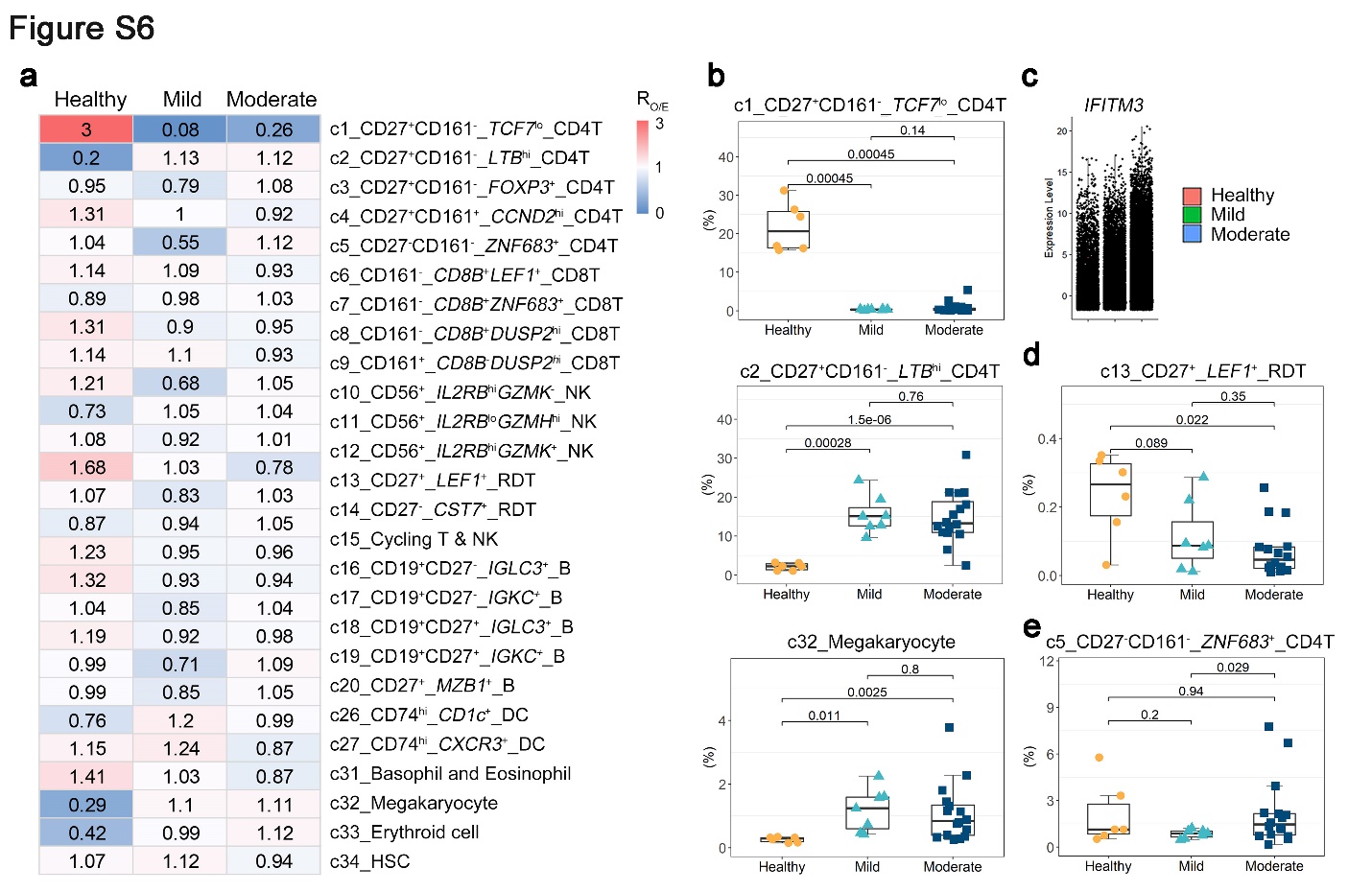


Figure S6. The changes in immune cell composition from Omicron convalescents and healthy donors.

**a.** The composition of immune cells other than monocytes and LDNs measured by the ratio of observed to randomly expected cell numbers (R_O/E_) in PBMCs from the Omicron convalescents (*n* = 7 for mild and *n* = 16 for moderate) and healthy donors (*n* = 6).

**b.** The differences of T cell clusters and megakaryocytes measured by percentages in PBMCs from the Omicron convalescents and healthy donors. *t*-test with Welch’s correction.

**c.** The expression of *IFITM3* in all monocyte clusters of Omicron convalescents with mild or moderate symptoms and healthy donors.

**d.** The differences of γδT cells measured by percentages in PBMCs from the moderate convalescents and healthy donors. *t*-test with Welch’s correction.

**e.** The differences of T cells measured by percentages in PBMCs from the mild and moderate convalescents. *t*-test with Welch’s correction.


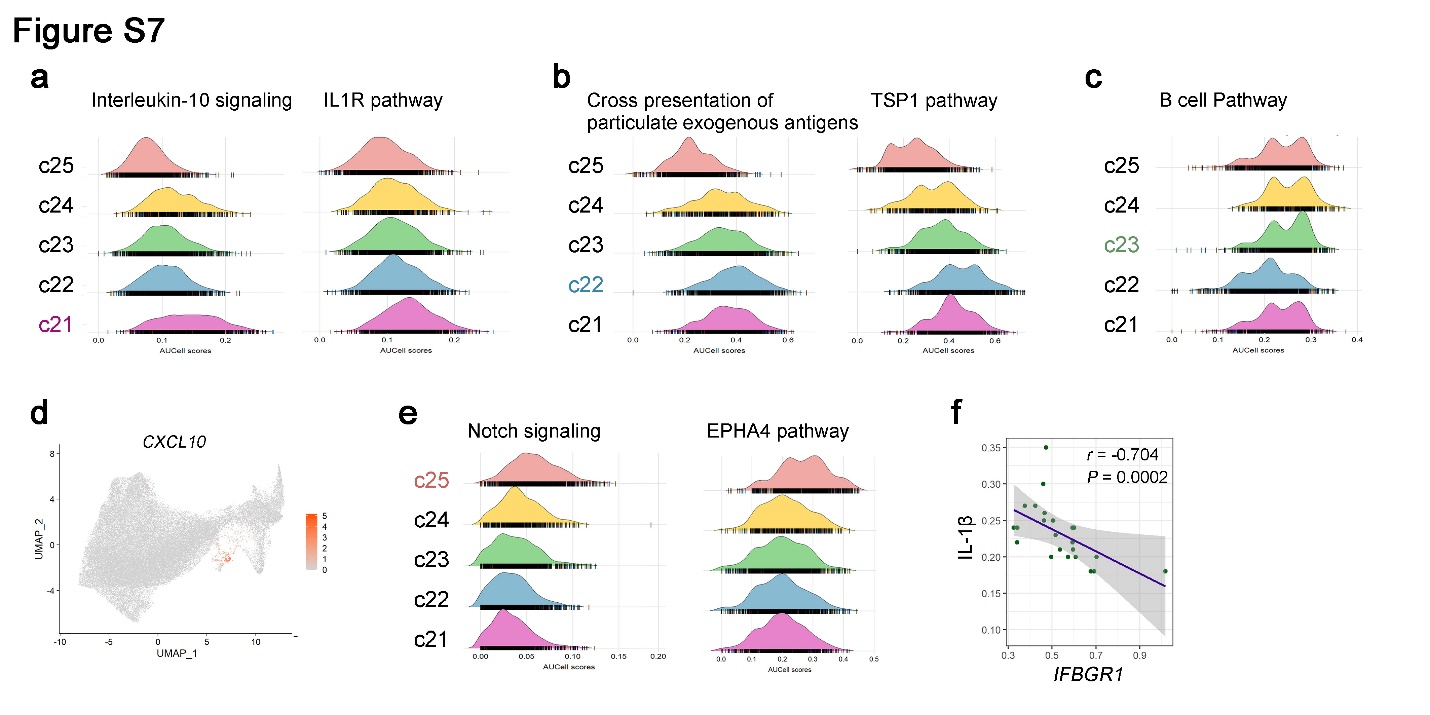


Figure S7. The expression of signature genes of monocyte clusters in Omicron convalescent individuals (*n* = 18) measured by WTA scRNA-seq.

**a.** AUcell plots showing the enrichment on the responses to IL-10 and IL1R signaling pathways by all monocyte clusters.

**b.** AUcell plots showing the enrichment on cross presentation of particulate exogenous antigens and TSP1 pathway by all monocyte clusters.

**c.** AUcell plots showing the enrichment on B cell activation by all monocyte clusters.

**d.** UMAP plots showing the cellular source of *CXCL10* in monocyte clusters.

**e.** AUcell plots showing the enrichment on the responses to NOTCH, and EPHA4-mediated signaling pathways by all monocyte clusters.

**f.** The correlation analysis on the level of plasma IL-1β and the expression of *IFNGR1* transcripts in monocytes from convalescents by scRNA-seq.


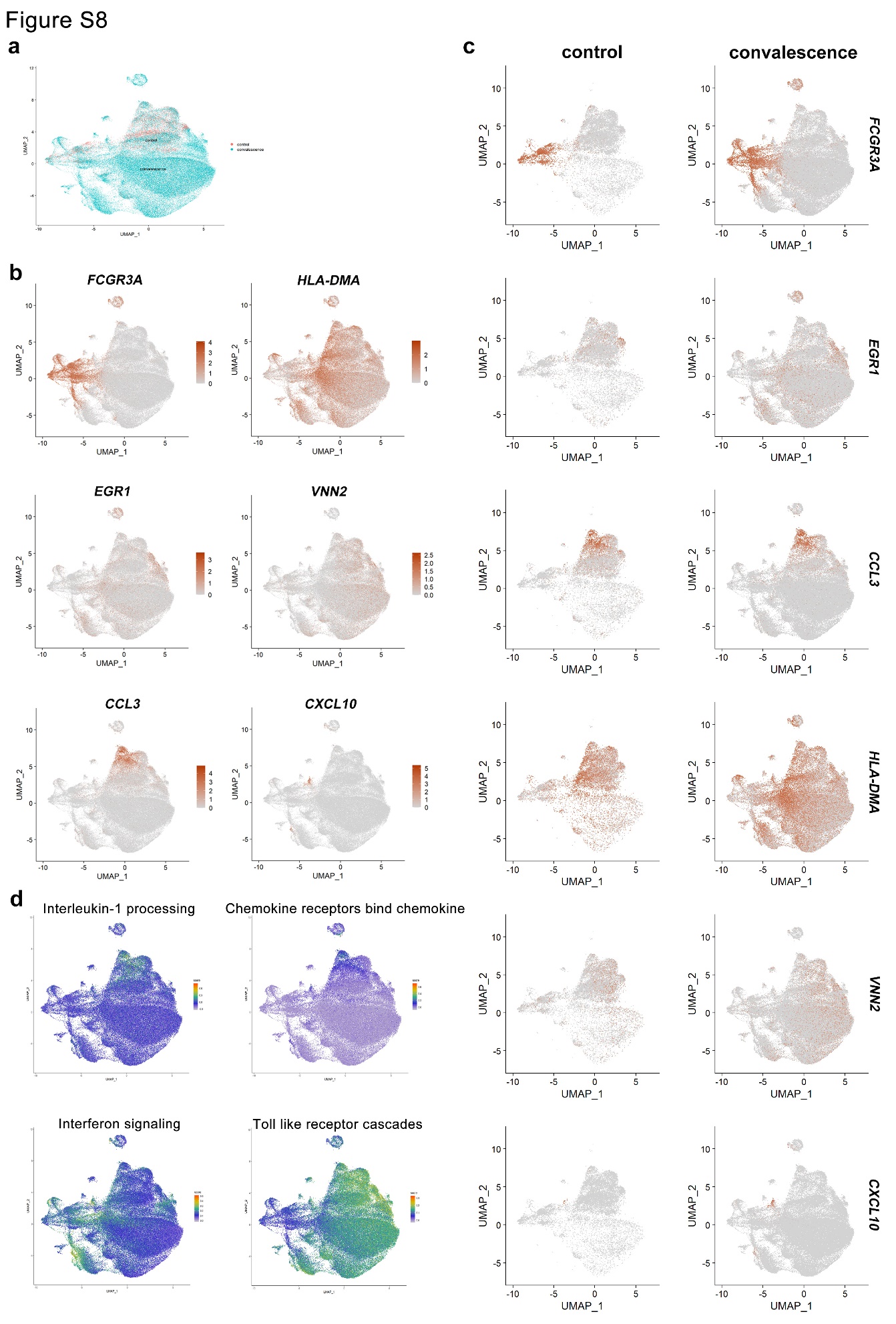


Figure S8. The monocyte composition and expression signature in PBMC from healthy donors and COVID-19 convalescents.

**a.** UMAP showing the distribution of monocyte clusters of 90,700 single cells from healthy donors (*n* = 15) and convalescents (*n*= 41) in the scRNA-seq dataset by Ren *et al.* (2021).[^11^](#_ENREF_11)

**b.** UMAP plots showing the cellular distribution of marker genes.

**c.** UMAP plots showing the differential cellular distribution of marker genes in convalescent and controls.

**d.** UMAP plots showing the enrichment on signaling pathways by monocyte clusters.


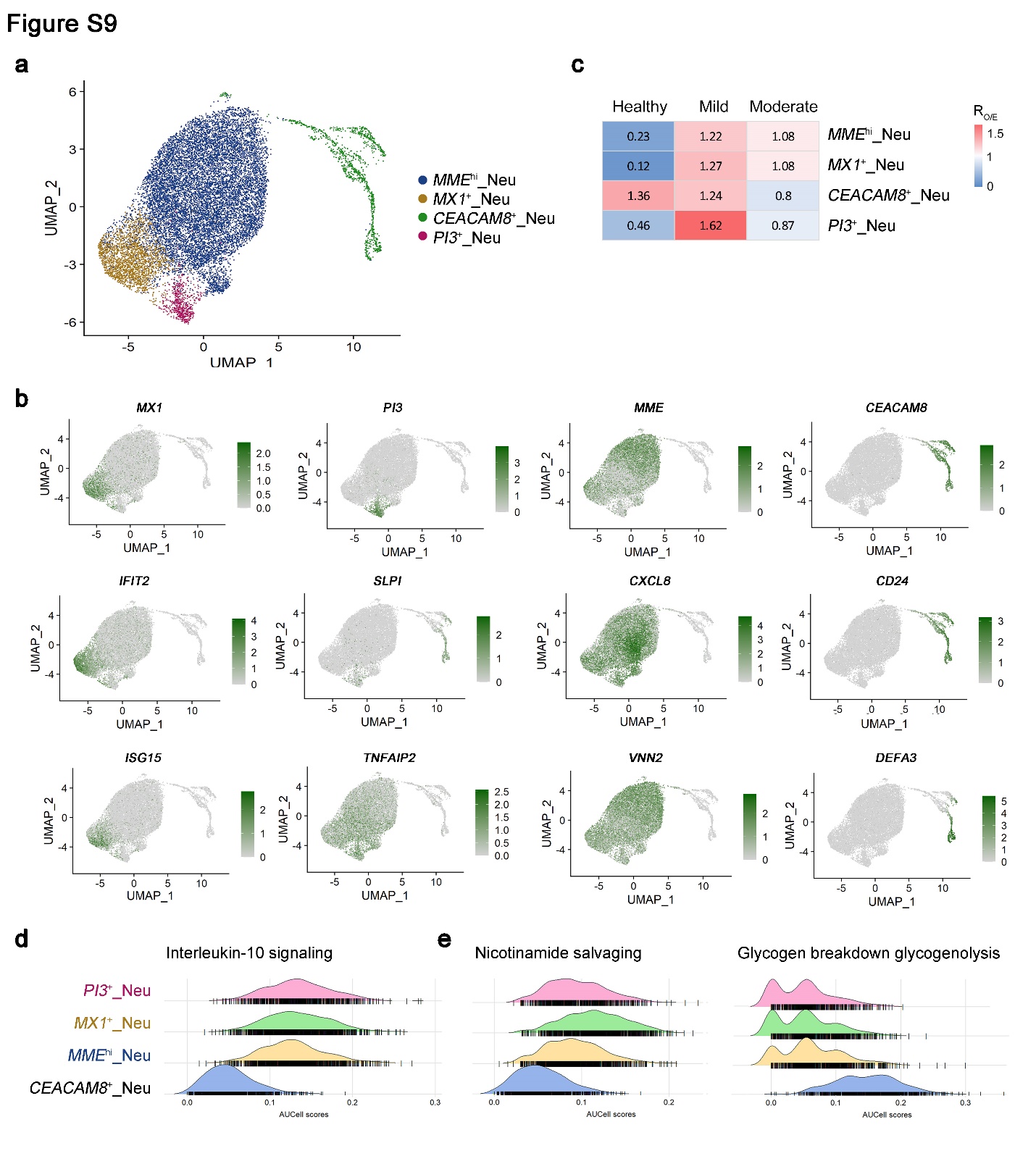


Figure S9. The expression of signature genes of LDN clusters and their composition in healthy and Omicron convalescent individuals measured by WTA scRNA-seq.

**a.** UMAP plots showing the cell clusters of LDNs from Omicron convalescents (*n* = 18) and healthy donors (*n* = 6) using data of WTA scRNA-seq.

**b.** UMAP plots showing the hallmarks or genes with abundant expression of LDN clusters measured by WTA scRNA-seq.

**c.** The preferences of LDN clusters measured by WTA scRNA-seq in healthy and convalescent individuals with distinct disease severity.

**d.** AUcell plots showing the enrichment on the responses to IL-10 signaling pathways by all the LDN clusters.

**e.** AUcell plots showing the enrichment on the metabolic processes by all the LDN clusters.


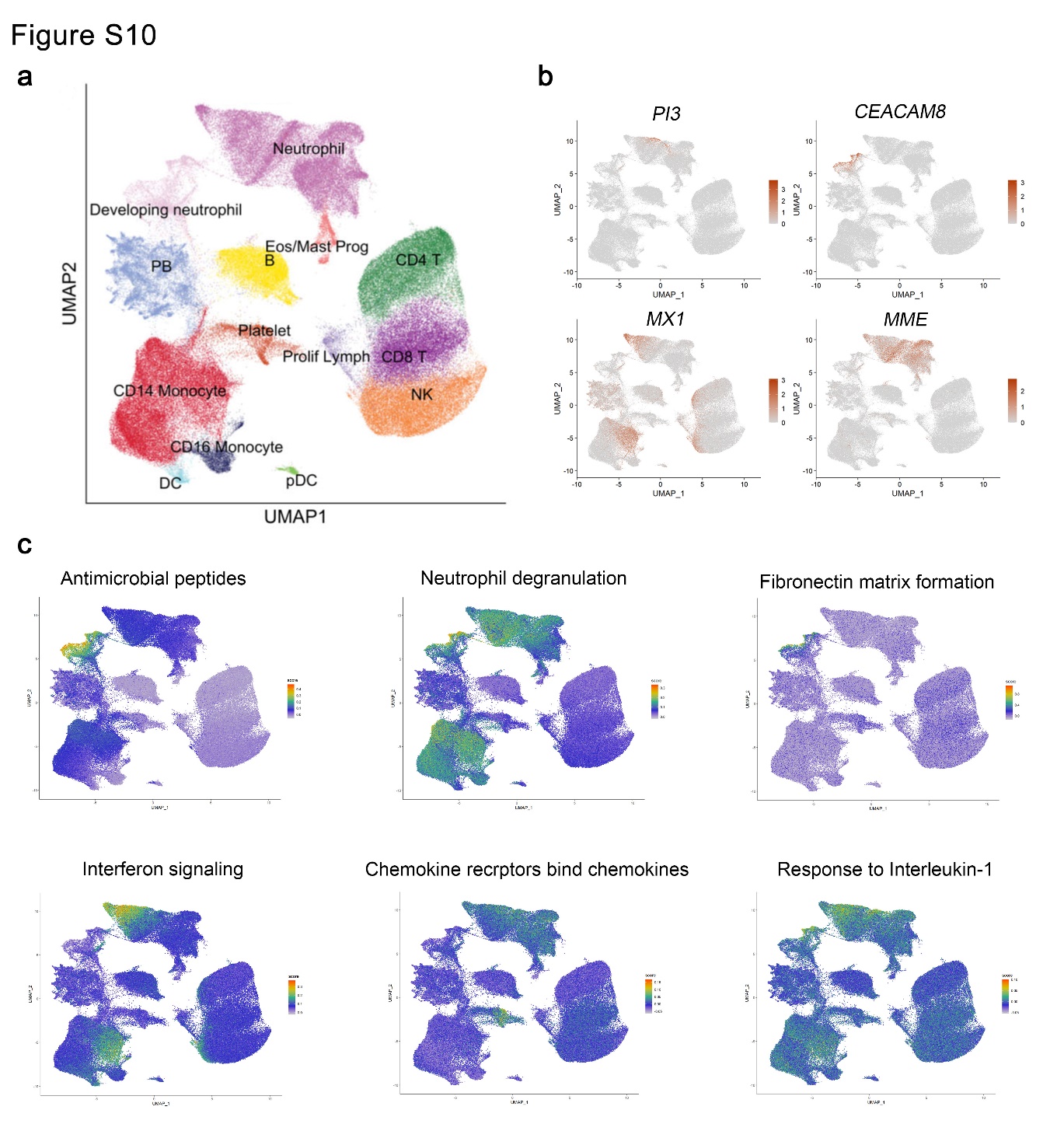


Figure S10. The neutrophil composition and expression signature in PBMC from COVID-19 patients infected with SARS-CoV-2 ancestral strain.

**a.** UMAP showing the distribution of cell types from the scRNA-seq dataset (cited from Fig 1D of the research article by Wilk, *et al.* (2021),[^12^](#_ENREF_12) including 174,753 cells from 25 acute SARS-CoV-2 samples, 7 convalescent SARS-CoV-2 samples and 8 healthy samples.

**b.** UMAP plots showing the cellular distribution of marker genes.

**c.** UMAP plots showing the enrichment on signaling pathways by neutrophil clusters.


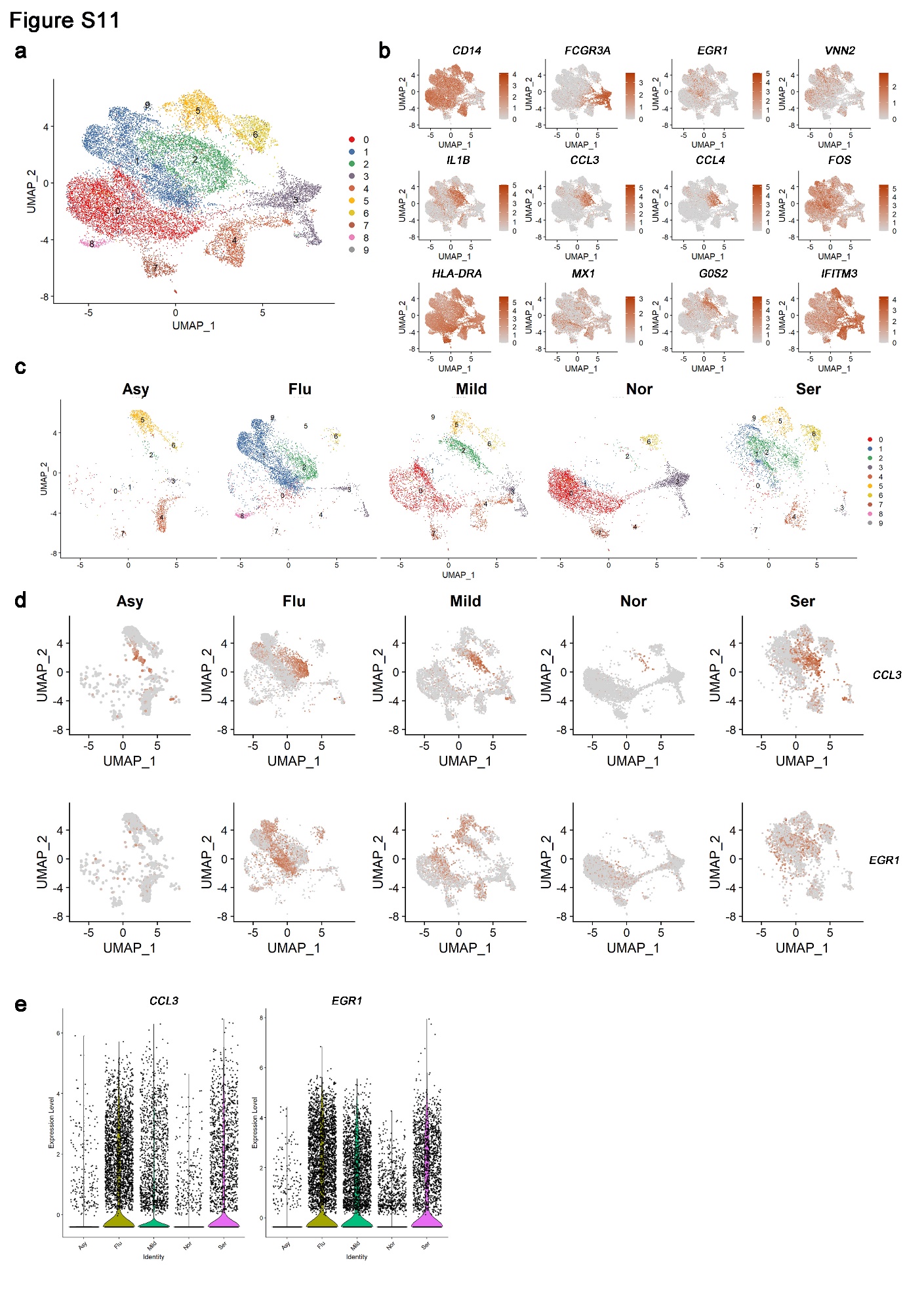


Figure S11. The expression of *EGR1* and *CCL3* in monocyte clusters from patients in the acute phase of infection using published data.

**a.** UMAP plots showing the monocyte clusters from the scRNA-seq dataset by Lee *et al.* (2020).[^13^](#_ENREF_13)

**b.** UMAP visualization of the immune features of the monocyte clusters.

**c.** The cellular composition of monocytes in healthy and patients.

**d.** UMAP visualization of the expression of *EGR1* and *CCL3* between different groups.

**e.** Violin plots visualization of the expression of *EGR1* and *CCL3* between different groups.


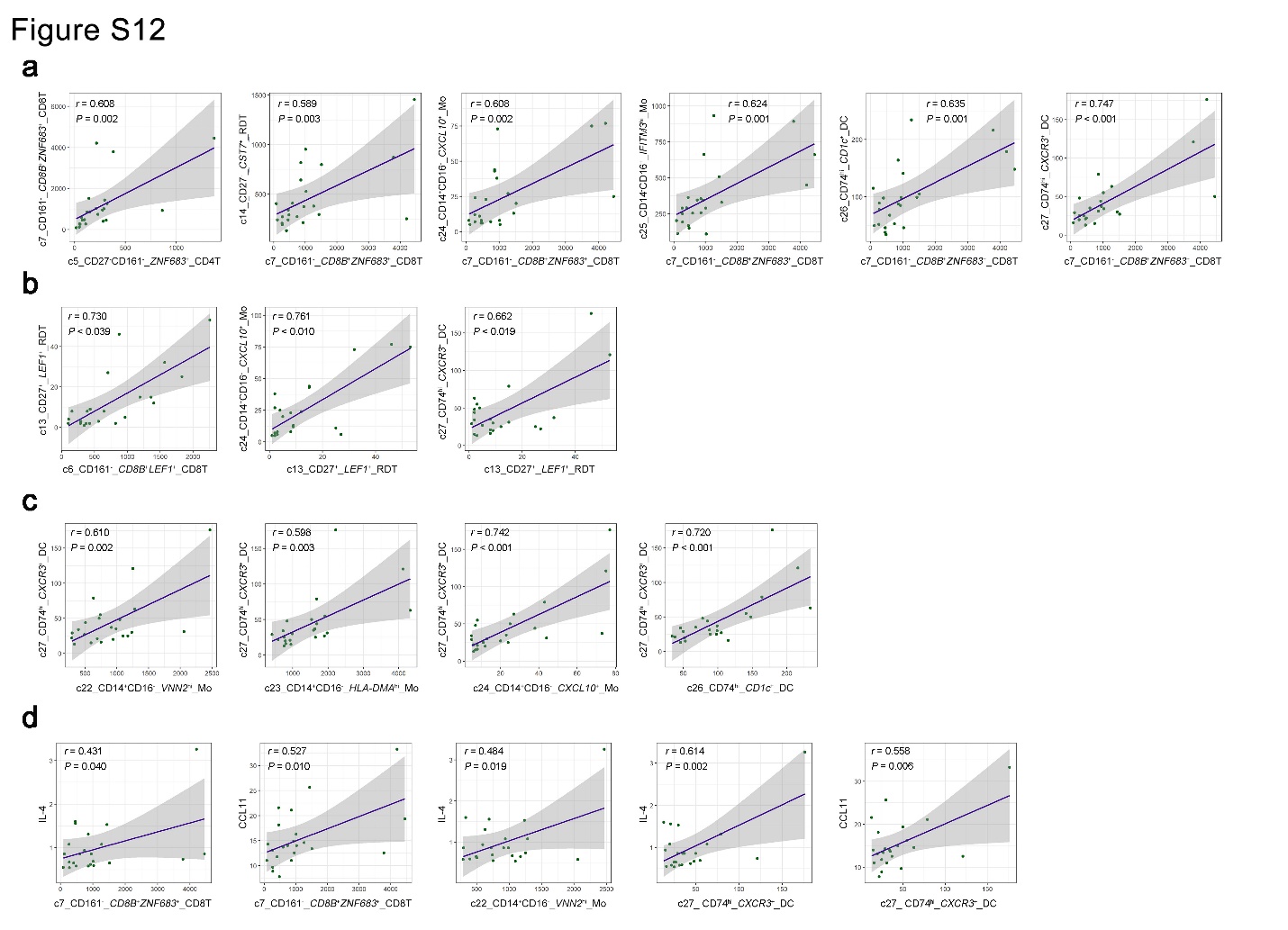


Figure S12. Association of immune cells and cytokines/chemokines in Omicron convalescents.

**a.** The correlation of immune cell clusters and c7_CD161^-^_*CD8B*^+^*ZNF683*^+^_CD8 T cells

in Omicron convalescents (*n* = 23).

**b.** The correlation of immune cell clusters and c13_ CD27^+^_*LEF*^+^_RDT cells

in Omicron convalescents (*n* = 23).

**c.** The correlation of immune cell clusters and c27_ CD74^hi^_*CXCR3*^+^_DCs in Omicron convalescents (*n* = 23).

**d.** The correlation of immune cell clusters and plasma IL-4 or CCL11 in Omicron convalescents (*n* = 23).

Table S1. Demographic characteristics and vaccination of SARS-COV-2 Omicron BA.1 convalescents and healthy donors involved in cytokine quantification.^*^

| **Characteristics** | **Mild convalescents**  **(*n* = 62)** | **Moderate convalescents**  **(*n* = 81)** | **Healthy donors**  **(*n* = 48)** |
| --- | --- | --- | --- |
| **Age — yr** | 38 (32-52) | 47 (36-57) | 43 (34-53) |
| **Gender** |  |  |  |
| Male | 27 (43.5%) | 39 (48.1%) | 29 (60.4%) |
| Female | 35 (56.5%) | 42 (51.9%) | 19 (39.6%) |
| **Vaccination ^#^** |  |  |  |
| 3 Doses of IV | 36 (58.1%) | 43 (53.1%) | 29 (60.4%) |
| 2 Doses of IV | 19 (30.6%) | 20 (24.7%) | 2 (4.2%) |
| 2 Doses of AVV | 4 (6.5%) | 12 (14.8%) | 17 (35.4%) |
| 1 Dose of AVV | 2 (3.2%) | 3 (3.7%) | - |
| Unvaccinated | 1 (1.6%) | 3 (3.7%) | - |

^*^ Continuous variables were shown in median (interquartile ranges) and categorical variables were summarized as counts (percentages).

^#^ IV, inactivated vaccine; AVV, adenovirus-vectored vaccine.

Table S2. Demographic characteristics and vaccination of SARS-COV-2 Omicron BA.1 convalescents and healthy donors involved in TTA scRNA-seq analysis.^*^

| **Characteristics** | **Mild convalescents**  **(*n* = 7)** | **Moderate convalescents**  **(*n* = 16)** | **Healthy donors**  **(*n* = 6)** |
| --- | --- | --- | --- |
| **Age — yr** | 30 (27-35) | 48 (36-56) | 46 (35-54) |
| **Gender** |  |  |  |
| Male | 4 (57.1%) | 7 (43.8%) | 3 (50.0%) |
| Female | 3 (42.9%) | 9 (56.3%) | 3 (50.0%) |
| **Vaccination ^#^** |  |  |  |
| 3 Doses of IV | 3 (42.9%) | 7 (43.8%) | 2 (33.3%) |
| 2 Doses of IV | 4 (57.1%) | 4 (25.0%) | 2 (33.3%) |
| 2 Doses of AVV | - | 5 (31.3%) | 2 (33.3%) |

^*^ Continuous variables were shown in median (interquartile ranges) and categorical variables were summarized as counts (percentages). Percentages may not total 100 because of rounding.

^#^ IV, inactivated vaccine; AVV, adenovirus-vectored vaccine.

Table S3. Demographic characteristics and vaccination of SARS-COV-2 Omicron BA.1 convalescents and healthy donors involved in WTA scRNA-seq analysis.^*^

| **Characteristics** | **Mild convalescents**  **(*n* = 5)** | **Moderate convalescents**  **(*n* = 13)** | **Healthy donors**  **(*n* = 6)** |
| --- | --- | --- | --- |
| **Age — yr** | 29 (25-30) | 47 (35-56) | 46 (35-54) |
| **Gender** |  |  |  |
| Male | 3 (60.0%) | 5 (38.5%) | 3 (50.0%) |
| Female | 2 (40.0%) | 8 (61.55) | 3 (50.0%) |
| **Vaccination ^#^** |  |  |  |
| 3 Doses of IV | 2 (40.0%) | 6 (46.2%) | 2 (33.3%) |
| 2 Doses of IV | 3 (60.0%) | 3 (23.1%) | 2 (33.3%) |
| 2 Doses of AVV | - | 4 (30.8%) | 2 (33.3%) |

^*^ Continuous variables were shown in median (interquartile ranges) and categorical variables were summarized as counts (percentages). Percentages may not total 100 because of rounding.

^#^ IV, inactivated vaccine; AVV, adenovirus-vectored vaccine.

Table S4. The peripheral immune cell composition of healthy donors and Omicron breakthrough convalescents.*

| **Major cell type** | **Cluster Name** | **Hallmark or characteristic gene expression** | | **Subset or cellular state** |
| --- | --- | --- | --- | --- |
|  |  | **Protein level** | **Transcript level** |  |
| CD4^+^ T | CD27^+^CD161^-^_*TCF7*^lo^_CD4T | CD27^+^CD28^+^CD161^-^ | *LEF*^hi^*TCF7*^lo^ | Resting or naive |
|  | CD27^+^CD161^-^_*LTB*^hi^_CD4T | CD27^+^CD28^+^CD161^-^ | *TCF7*^hi^*LTB*^hi^ | Effector |
|  | CD27^+^CD161^-^_*FOXP3*^+^_CD4T | CD27^+^CD28^+^CD161^-^ | *FOXP3*^+^*RGS1*^+^ | Regulatory T cells |
|  | CD27^+^CD161^+^_*CCND2*^hi^_CD4T | CD27^+^CD28^+^CD161^+^ | *LTB*^hi^*CCND2*^hi^ | Activated and stem-like |
|  | CD27^-^CD161^-^_*ZNF683*^+^_CD4T | CD27^-^CD28^-^CD161^-^ | *ZNF683*^+^ | Cytotoxic T cells |
| CD8^+^ T | CD161^-^_*CD8B*^+^*LEF1*^+^_CD8T | CD8A^+^CD161^-^ | *CD8B*^+^*LEF1*^+^*CCR7*^+^ | Resting or naive, low cytotoxic |
|  | CD161^-^_*CD8B*^+^*ZNF683*^+^_CD8T | CD8A^+^CD161^-^ | *CD8B*^+^*ZNF683*^+^*GZMH*^+^ | Effector/memory, GZMH cytotoxic |
|  | CD161^-^_*CD8B*^+^*DUSP2*^hi^_CD8T | CD8A^+^CD161^-^ | *CD8B*^+^*DUSP2*^hi^*GZMK*^+^ | Activated, GZMK cytotoxic |
|  | CD161^+^_*CD8B*^-^*DUSP2*^hi^_CD8T | CD8A^+^CD161^+^ | *CD8B*^-^*DUSP2*^hi^*GZMK*^+^ | Activated, GZMK cytotoxic, (MAIT) |
| NK cells | CD56^+^_*IL2RB*^hi^*GZMK*^-^_NK | CD56^+^ | *IL2RB*^hi^*GZMK*^-^*GZMH*^mid^*DUSP2*^lo^*CST7*^hi^ | Low activated, GZMH cytotoxic |
|  | CD56^+^_*IL2RB*^lo^*GZMH*^hi^_NK | CD56^+^ | *IL2RB*^lo^*GZMK*^-^*GZMH*^hi^*DUSP2*^lo^*CST7*^hi^ | Low activated, GZMH cytotoxic |
|  | CD56^+^_*IL2RB*^hi^*GZMK*^+^_NK | CD56^+^ | *IL2RB*^hi^*GZMK*^+^*GZMH*^lo^*DUSP2*^hi^*CST7*^lo^ | Activated, GZMK cytotoxic |
| TCR_γδ_ T | CD27^+^_*LEF1*^+^_RDT | CD27^+^ | *LEF1*^+^*DUSP2*^lo^ | Resting, low cytotoxic |
|  | CD27^-^_*CST7*^+^_RDT | CD27^-^ | *CST7*^+^*DUSP2*^hi^*PRF1*^+^*GZMH*^+^ | Activated and cytotoxic |
| Cycling T | Cycling T & NK | N/A | *TYMS*^+^*PCNA*^+^*KIAA0101*^+^ | Proliferative |
| B cells | CD19^+^CD27^-^_*IGLC3*^+^_B | CD19^+^CD27^-^ | *IGLC3*^hi^*IG-membrane*^hi^ | Resting, mature B (λ) |
|  | CD19^+^CD27^-^_*IGKC*^+^_B | CD19^+^CD27^-^ | *IGKC*^hi^*IG-membrane*^hi^ | Resting, mature B(κ) |
|  | CD19^+^CD27^+^_*IGLC3*^+^_B | CD19^+^CD27^+^ | *IGLC3*^hi^*IG-membrane*^lo^*IG-secreted*^mid^ | Activated, memory B (λ) |
|  | CD19^+^CD27^+^_*IGKC*^+^_B | CD19^+^CD27^+^ | *IGKC*^hi^*IG-membrane*^lo^*IG-secreted*^mid^ | Activated, memory B cells (κ) |
|  | CD27^+^_*MZB1*^+^_B | CD27^+^ | *MZB1*^+^*IG-secreted*^hi^ | Plasma cells |
| Monocytes | CD14^+^CD16^-^_*EGR1*^+^_Mo | CD14^+^CD16^-^ | *EGR1*^+^ | Naive and resting |
|  | CD14^+^CD16^-^_*VNN2*^hi^_Mo | CD14+CD16- | *VNN2*^hi^ | Suppressive |
|  | CD14^+^CD16^-^_*HLA-DMA*^hi^_Mo | CD14^+^CD16^-^ | *HLA-DMA*^hi^ | Activated |
|  | CD14^+^CD16^-^_*CXCL10*^+^_Mo | CD14^+^CD16^-^ | *HLA-DMA*^hi^*CXCL10*^+^*IFITM3*^hi^ | Activated, IFN-responsive |
|  | CD14^-^CD16^+^_*IFITM3*^hi^_Mo | CD14^-^CD16^+^ | *IFITM3*^hi^ | Activated, IFN-responsive |
| DC | CD74^hi^_*CD1c*^+^_DC | CD74^hi^ | *CD1c*^+^*CLEC10A*^+^*FLT3*^+^ | CD1c^+^ DC |
|  | CD74^hi^_*CXCR3*^+^_DC | CD74^hi^ | *CXCR3*^+^*IRF4*^+^*FLT3*^+^ | cDC and pDC |
| Low-density Neutrophil (LDN) | CD16^hi^CD127^hi^_*MME*^hi^_Neu | CD16^hi^CD127^hi^ | *CXCR2*^+^*CMTM2*^+^*MME*^hi^ | Low-density |
|  | CD16^hi^CD127^hi^_*PI3*^+^_Neu | CD16^hi^CD127^hi^ | *CXCR2*^+^*CMTM2*^+^*PI3*^+^ | Low-density |
|  | CD16^lo^CD127^lo^_*CEACAM8*^+^_Neu | CD16^lo^CD127^lo^ | *CEACAM8*^+^ | Low-density, immature |
| Basophil and Eosinophil | Basophil and Eosinophil | N/A | *CLC*^+^*CPA3*^+^ | N/A |
| Megakaryocyte | Megakaryocyte | N/A | *F13A1*^hi^ | N/A |
| Erythroid cell | Erythroid cell | N/A | *ALAS2*^hi^ | N/A |
| HSC | HSC | N/A | *CD34*^+^ | N/A |

* Cell clusters were named in the order of surface antigens at protein levels, then genes at transcript level followed by major cell types. Genes or proteins expressed or non-expressed in some clusters were indicated as positive (+) or negative (-) and expressed at high, middle or low levels in some clusters were indicated as hi, mid and lo respectively. MAIT, mucosal-associated invariant T cells; N/A, not applied. See also Fig. 2a and Supplementary Fig. S3 and S4.

Table S5. Demographic characteristics and vaccination of SARS-CoV-2 Omicron BA.1 convalescents with PASC involved in cytokine quantification.^*^

| **Characteristics** | **With PASC**  **(*n* = 25)** | **Without PASC**  **(*n* = 118)** |
| --- | --- | --- |
| **Age — yr** | 46 (38-57) | 42 (34-56) |
| **Gender** |  |  |
| Male | 13 (52.0%) | 53 (44.9%) |
| Female | 12 (48.0%) | 65 (55.1%) |
| **Vaccination ^#^** |  |  |
| 3 Doses of IV | 17 (68.0%) | 62 (52.5%) |
| 2 Doses of IV | 5 (20.0%) | 34 (28.8%) |
| 2 Doses of AVV | 1 (4.0%) | 15 (12.7%) |
| 1 Dose of AVV | 1 (4.0%) | 4 (3.4%) |
| Unvaccinated | 1 (4.0%) | 3 (2.5%) |
| **COVID-19 severity** |  |  |
| Mild | 8 (32.0%) | 54 (45.8%) |
| Moderate | 17 (68.0%) | 64 (54.2%) |
| **PASC symptoms ^^^** |  |  |
| General symptoms | 10 (40.0%) | - |
| Respiratory symptoms | 18 (72.0%) | - |
| Digestive symptoms | 8 (32.0%) | - |
| Neurological symptoms | 10 (40.0%) | - |
| Skin and mucous membrane symptoms | 11 (44.0%) | - |
| Circulation symptoms | 9 (36.0%) | - |

^*^ Continuous variables were shown in median (interquartile ranges) and categorical variables were summarized as counts (percentages). Percentages may not total 100 because of rounding.

^#^ IV, inactivated vaccine; AVV, adenovirus-vectored vaccine.

^^^ Some patients have two or more symptoms of PASC. General symptoms: fatigue, fever; Respiratory symptoms: cough, nasal congestion, nasal discharge, sore throat; Digestive symptoms: diarrhoea; Neurological symptoms: allotriogeusia, heterosmia; Skin and mucous membrane symptoms: rash, conjunctivitis, mucosal inflammation; and Circulation symptoms: hypotension.

Table S6. Demographic characteristics and vaccination of SARS-CoV-2 Omicron BA.1 convalescents with PASC involved in scRNA-seq.^*^

| **Characteristics** | **With PASC**  **(*n* = 4)** | **Without PASC**  **(*n* = 19)** |
| --- | --- | --- |
| **Age — yr** | 57 (49-57) | 36 (32-51) |
| **Gender** |  |  |
| Male | 3 (75.0%) | 8 (42.1%) |
| Female | 1 (25.0%) | 11 (57.9%) |
| **Vaccination ^#^** |  |  |
| 3 Doses of IV | 2 (50.0%) | 8 (42.1%) |
| 2 Doses of IV | 2 (50.0%) | 6 (31.6%) |
| 2 Doses of AVV | 0 (0.0%) | 5 (26.3%) |
| **COVID-19 severity** |  |  |
| Mild | 1 (25.0%) | 6 (31.6%) |
| Moderate | 3 (75.0%) | 13 (68.4%) |
| **PASC symptoms** ^^^ |  |  |
| General symptoms | 1 (25.0%) | - |
| Respiratory symptoms | 2 (50.0%) | - |
| Digestive symptoms | 0 (0.0%) | - |
| Neurological symptoms | 1 (25.0%) | - |
| Skin and mucous membrane symptoms | 1 (25.0%) | - |
| Circulation symptoms | 0 (0.0%) | - |

^*^ Continuous variables were shown in median (interquartile ranges) and categorical variables were summarized as counts (percentages). Percentages may not total 100 because of rounding.

^#^ IV, inactivated vaccine; AVV, adenovirus-vectored vaccine.

^^^ Some patients have two or more symptoms of PASC. General symptoms: fatigue, fever; Respiratory symptoms: cough, nasal congestion, nasal discharge, sore throat; Digestive symptoms: diarrhoea; Neurological symptoms: allotriogeusia, heterosmia; Skin and mucous membrane symptoms: rash, conjunctivitis, mucosal inflammation; and Circulation symptoms: hypotension.

Table S7. COVID-19 severity, vaccination and symptoms of Omicron convalescents with PASC involved in scRNA-seq.

|  | **Convalescent 1** | **Convalescent 2** | **Convalescent 3** | **Convalescent 4** |
| --- | --- | --- | --- | --- |
| **Age** | 57 | 27 | 56 | 58 |
| **Gender** | Male | Male | Female | Male |
| **COVID-19 severity** | Mild | Moderate | Moderate | Moderate |
| **Vaccination^*^** | IV2 | IV2 | IV3 | IV3 |
| **PASC symptoms** | Allotriogeusia  and diarrhoea | Cough | Fever | Sore throat |
| **Cell counts of clusters** |  |  |  |  |
| c7 | 4202 | 3788 | 457 | 1436 |
| c13 | 46 | 53 | 3 | 9 |
| c27 | 176 | 121 | 13 | 30 |

^*^ IV2, 2 doses of inactivated vaccines; IV3, 3 doses of inactivated vaccines.

Table S8. List of BD® AbSeq Immune Discovery Panel.

| **Specificity** | **Clone** | **Oligo ID** |
| --- | --- | --- |
| CD3 | UCHT1 | AHS0231 |
| CD4 | SK3 | AHS0032 |
| CD8 | SK1 | AHS0228 |
| CD11c | B-Ly6 | AHS0056 |
| CD14 | MPHIP9 | AHS0037 |
| CD16 | 3G8 | AHS0053 |
| CD19 | SJ25C1 | AHS0030 |
| CD25 | 2A3 | AHS0026 |
| CD27 | M-T271 | AHS0025 |
| CD28 | L293 | AHS0138 |
| CD45RA | HI100 | AHS0009 |
| CD56 | NCAM16 | AHS0019 |
| CD62L | DREG-56 | AHS0049 |
| CD127 | HIL-7R-M21 | AHS0028 |
| CD134 | ACT35 | AHS0013 |
| CD137 | 4B4-1 | AHS0003 |
| CD161 | HP-3G10 | AHS0205 |
| CD183 (CXCR3) | 1C6/CXCR3 | AHS0031 |
| CD185 (CXCR5) | RF8B2 | AHS0039 |
| CD186 (CXCR6) | 13B 1E5 | AHS0148 |
| CD196 (CCR6) | 11A9 | AHS0034 |
| CD197 (CCR7) | 2-L1-A | AHS0273 |
| CD272 | J168-540 | AHS0052 |
| CD278 | DX29 | AHS0012 |
| CD279 | EH12.1 | AHS0014 |
| CD357 (GITR) | V27-580 | AHS0104 |
| CD366 (TIM-3) | 7D3 | AHS0016 |
| HLA-DR | G46-6 | AHS0035 |
| IgD | IA6-2 | AHS0058 |
| IgM | G20-127 | AHS0198 |

References

1 Zheng, H. *et al.* Disease profile and plasma neutralizing activity of post-vaccination Omicron BA.1 infection in Tianjin, China: a retrospective study. *Cell research*, doi:10.1038/s41422-022-00674-2 (2022).

2 World Health Organization. *Living guidance for clinical management of COVID-19: living guidance, 23 November 2021*, <https://apps.who.int/iris/handle/10665/349321> (2021).

3 Lombardelli, L., Logiodice, F., Kullolli, O. & Piccinni, M. P. Evaluation of Secreted Cytokines by Multiplex Bead-Based Assay (X MAP Technology, Luminex). *Methods in molecular biology* **2285**, 121-130, doi:10.1007/978-1-0716-1311-5_10 (2021).

4 Mair, F. *et al.* A Targeted Multi-omic Analysis Approach Measures Protein Expression and Low-Abundance Transcripts on the Single-Cell Level. *Cell reports* **31**, 107499, doi:10.1016/j.celrep.2020.03.063 (2020).

5 Hao, Y. *et al.* Integrated analysis of multimodal single-cell data. *Cell* **184**, 3573-3587 e3529, doi:10.1016/j.cell.2021.04.048 (2021).

6 McGinnis, C. S., Murrow, L. M. & Gartner, Z. J. DoubletFinder: Doublet Detection in Single-Cell RNA Sequencing Data Using Artificial Nearest Neighbors. *Cell systems* **8**, 329-337 e324, doi:10.1016/j.cels.2019.03.003 (2019).

7 Hafemeister, C. & Satija, R. Normalization and variance stabilization of single-cell RNA-seq data using regularized negative binomial regression. *Genome biology* **20**, 296, doi:10.1186/s13059-019-1874-1 (2019).

8 Aibar, S. *et al.* SCENIC: single-cell regulatory network inference and clustering. *Nature methods* **14**, 1083-1086, doi:10.1038/nmeth.4463 (2017).

9 Liberzon, A. *et al.* The Molecular Signatures Database (MSigDB) hallmark gene set collection. *Cell systems* **1**, 417-425, doi:10.1016/j.cels.2015.12.004 (2015).

10 Bergen, V., Lange, M., Peidli, S., Wolf, F. A. & Theis, F. J. Generalizing RNA velocity to transient cell states through dynamical modeling. *Nature biotechnology* **38**, 1408-1414, doi:10.1038/s41587-020-0591-3 (2020).

11 Ren, X. *et al.* COVID-19 immune features revealed by a large-scale single-cell transcriptome atlas. *Cell* **184**, 1895-1913 e1819, doi:10.1016/j.cell.2021.01.053 (2021).

12 Wilk, A. J. *et al.* Multi-omic profiling reveals widespread dysregulation of innate immunity and hematopoiesis in COVID-19. *The Journal of experimental medicine* **218**, doi:10.1084/jem.20210582 (2021).

13 Lee, J. S. *et al.* Immunophenotyping of COVID-19 and influenza highlights the role of type I interferons in development of severe COVID-19. *Science immunology* **5**, doi:10.1126/sciimmunol.abd1554 (2020).
